# Macrophages Internalize Epithelial‐Derived Extracellular Vesicles That Contain Ferritin via the Macrophage Scavenger Receptor 1 to Promote Inflammatory Bowel Disease

**DOI:** 10.1002/jev2.70105

**Published:** 2025-06-23

**Authors:** Wenxin Zhang, Weichen Dong, Chen Cheng, Hongting Zhao, Yutong Liu, Kristina Zaydel, Liora Frech, Zhiyao Xing, Wusheng Zhu, Bing Zhou, Yi Li, Esther G. Meyron‐Holtz, Kuanyu Li

**Affiliations:** ^1^ State Key Laboratory of Pharmaceutical Biotechnology, Department of Vascular Surgery, Nanjing Drum Tower Hospital, Affiliated Hospital of Medical School, Jiangsu Key Laboratory of Molecular Medicine, Medical School Nanjing University Nanjing China; ^2^ State Key Laboratory of Pharmaceutical Biotechnology, Department of Neurology, Nanjing Jinling Hospital, Affiliated Hospital of Medical School Nanjing University Nanjing China; ^3^ Laboratory of Molecular Nutrition, Department of Biotechnology and Food Engineering Technion–Israel Institute of Technology Haifa Israel; ^4^ Faculty of Synthetic Biology Shenzhen University of Advanced Technology Shenzhen China; ^5^ Department of General Surgery, Nanjing Jinling Hospital, Affiliated Hospital of Medical School Nanjing University Nanjing China

**Keywords:** extracellular vesicles, ferritin trafficking, IBD, intestinal epithelial cells, macrophages, macrophage scavenger receptor 1

## Abstract

The incidence of inflammatory bowel disease (IBD) is on the rise, yet current clinical treatments are limited. Previous studies have identified impairments in both systemic and local iron metabolism in IBD patients. However, the impact of iron dyshomeostasis on the development and pathogenesis of IBD remains elusive. In this study, we confirmed iron deposition in inflamed intestinal lesions of IBD patients and mice with DSS‐induced colitis, accompanied by distinct distribution patterns of the iron storage protein ferritin. To reveal the role of ferritin in the involvement of pathology of IBD, we constructed intestinal epithelial cell‐ or myeloid‐specific ferritin H (*FtH*) knockout mice and demonstrated that intestinal epithelial cells (IECs) release extracellular vesicles (EVs) that contain iron‐loaded ferritin. These EVs are internalized by macrophages via the macrophage scavenger receptor 1 (Msr1), leading to the activation of inflammatory responses and oxidative stress, thereby exacerbating colitis severity. Genetic deletion of *FtH* in IECs or blockage of macrophage ferritin uptake, either through Msr1 inhibitor fucoidan or through *Msr1* knockdown (KD), suppressed inflammatory symptoms. Thus, EVs containing iron‐loaded ferritin released from IECs activate macrophages and contribute to IBD development, supporting that IBD patients with iron deficiency anaemia are often prescribed iron supplementation in a remission phase, other than in an active phase of the disease. Pharmacological inhibition of this ferritin secretion and engulfing process provides a therapeutic target for the disease.

## Graphical abstract

This study explores the interaction between intestinal epithelial cells (IECs) and macrophages through extracellular vesicles (EVs) in the context of inflammatory bowel disease (IBD). IECs release iron‐loaded ferritin via EVs as the donor, while macrophage scavenger receptor 1 on the surface of macrophages serves as the receptor for the internalization of the iron‐loaded ferritin‐containing EVs. This process activates macrophages and contributes to the development of IBD. These findings highlight the pathophysiological significance of iron metabolism disorders and suggest potential targets for future clinical applications in patients with IBD.

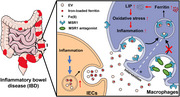

## 1 Introduction

Inflammatory bowel disease (IBD) encompasses complex, long‐term, recurrent gastrointestinal pathophysiology, including Crohn's disease (CD) and ulcerative colitis (UC), and has become increasingly prevalent in recent years (Kobayashi and Hibi 2023). The aetiology of IBD is still unclear, but it is believed that many hereditary and environmental factors mediate modifications of microbiota and abnormal immune function, leading to pathological inflammation and tissue damage, which contribute to the development of the disease (Cassinotti et al. 2014; Ocansey et al. 2020; Zhao et al. 2022). Additionally, patients with IBD are at a high risk of developing malignant diseases such as colorectal cancer, which seriously impacts their quality of life (Nadeem et al. 2020).

Iron metabolism is impaired in IBD patients (Oldenburg et al. 2001; Cacoub et al. 2022; Marques et al. 2022; Harrer et al. 2025). Clinical studies have shown a significant increase in the incidence of iron‐deficient anaemia among these patients, yet serum ferritin levels, which typically reflect systemic iron levels, are unusually high (Mecklenburg et al. 2014; Loveikyte et al. 2023). Significant iron deposits have been observed in inflamed intestinal tissues of IBD patients compared to their non‐inflamed tissues or healthy counterparts (Lih‐Brody et al. 1996; Harrer et al. 2025). Iron regulatory proteins (IRPs) are required to support a proper immune response but also contribute to inflammation (Cheng et al. 2024; Harrer et al. 2025). These paradoxical observations remain poorly understood, and the role of iron metabolism disorders in the development of IBD warrants further investigation.

Ferritin, a pivotal protein in iron homeostasis, is a 24‐mer nanocage composed of two subunits, the L‐subunit (FTL) and H‐subunit (FTH), with varying ratios in different tissues or cell types (Recalcati et al. 2008; Arosio et al. 2009). Beyond its classical iron storage function, ferritin involves other pathophysiological aspects, including inflammation and immunity (Wang et al. 2010; Mahroum et al. 2022). It is secreted for intercellular communications, reshaping the adjacent microenvironment and influencing neighbouring cells (Leichtmann‐Bardoogo et al. 2012; Mukherjee et al. 2020; Maass et al. 2021; Zhang et al. 2021). Extracellular vesicles (EVs) are likely a significant pathway for ferritin secretion (Cohen et al. 2010; Truman‐Rosentsvit et al. 2018). CD63, a common EV marker, is post‐transcriptionally regulated by IRP via the iron‐responsive element‐IRP (IRE‐IRP) system and is essential for ferritin secretion (Torti and Torti 2021; Yanatori et al. 2021). Secreted holo‐ferritin in EVs has been verified to function as an iron transporter or oxidative stress regulator in brain or milk (Palsa et al. 2023; Palsa et al. 2023). However, the specific cell types that secrete ferritin and the role of secreted ferritin in IBD remain largely unexplored.

In the present study, we confirmed aberrant iron distribution in the intestinal tracts of IBD patients and mice with colitis. We found that FTH in intestinal macrophages was largely derived from intestinal epithelial cells (IECs). The IECs secrete EV‐carried ferritin, which contains and transfers iron into macrophages. These macrophages recognize and internalize EVs by macrophage scavenger receptor 1 (MSR1). Consequently, intercellular ferritin trafficking contributes to iron redistribution, oxidative stress, and the progression of inflammation in IBD. Blocking this trafficking process alleviates IBD symptoms in mice. These findings enhance our understanding of the pathophysiological processes and may inform clinical strategies for treating IBD.

## 2 Methods

### 2.1 Participants

Participants in this study included 10 patients with bowel resection for ileal or colonic stricture CD, aged 18–65 years, recruited from the Department of General Surgery, Jinling Hospital, Affiliated Hospital of Nanjing University Medical School in 2023. Intestinal samples containing the injured segments and the resection margin were collected and separated into two parts: one snap‐frozen and stored at −80°C for further gene and protein analysis, and another fixed in 4% phosphate‐buffered formalin and embedded in paraffin for histological analysis. This study complies with the Declaration of Helsinki and was approved by the Institutional Review Board of Nanjing University Medical School (#OAP20230526001). Patients or the public were not involved in the design, conduct, reporting, or dissemination plans of our research. For detailed information on participants, see the Table S1.

### 2.2 Animals

All experiments were performed according to the NIH Guide for the Care and Use of Laboratory Animals and were approved by the Animal Experimentation Administration of Nanjing University (#IACUC‐D2202121). Mice *FtH*‐loxp (*FtH^fl/fl^*), *LysM^cre^* and *Vil^Cre^* with C57BL/6J background were purchased from Cyagen Bioscience (Suzhou, China). To obtain *FtH^LysM/LysM^* and *FtH^Vil/Vil^* mice, mating between *LysM^cre^* or *Vil^Cre^* mice and *FtH^fl/fl^* mice was carried out. Mouse DNA was extracted following instructions (Mouse Tissue Lysis Kit, APExBIO, Boston, MA). The primers for PCR genotyping are as follows:

*FtH*‐loxp: Forward: 5’‐CCATCAACCGCCAGATCAAC‐3’,
Reverse: 5’‐ CGCCATACTCCAGGAGGAAC‐3’.
*LysM^Cre^*: Forward: 5’‐CTTGGGCTGCCAGAATTTCTC‐3’,
Reverse: 5’‐CGCGCCTGAAGATATAGAAGA‐3’.
*Vil^Cre^*: Forward: 5’‐ GTGTTTGGTTTGGTTTCCTCTGCATAAGA‐3’,
Reverse: 5’‐ GCAGGCAAATTTTGGTGTACGGTCA‐3’.

The mice were fed a standard rodent diet (195 mg/kg iron, Xietong, Jiangsu, China), maintained in a constant 12‐h light/dark cycle, and had access to food and water ad libitum. For colonic epithelial ferritin expression in mice, an in vivo jetPEI system (Polyplus‐transfection, NY, USA) was used for plasmid delivery. Briefly, 0.2 mg Dendra2 or Dendra2‐FtH‐expressing plasmid was mixed with polyethyleneimine (PEI) and dissolved in 200 µL saline and slowly administered into the lumen of the colon via a catheter inserted about 4 cm into the colon through the anus. The mice were treated with dextran sulphate sodium (DSS) 72 h later.

### 2.3 Dextran Sulphate Sodium‐Induced Colitis Model and Tissue Collection

Male mice aged 7–10 weeks were randomly divided into sham and colitis groups. For the colitis group, DSS with an average MW of 36,000–50,000 Da (MP Biomedicals, Cat# 9011‐18‐1, Solon, OH, USA) was added to sterile water at a concentration of 2.5% and administered ad libitum for 7 days. For the sham group, mice drank sterile water all the time. The distal colon was collected and washed softly with a cold phosphate‐buffered saline (PBS) solution. One half of the tissue was fixed in 4% polyformaldehyde for histological experiments, and another half was quickly frozen in liquid nitrogen and then stored in a −80°C freezer for the extraction of RNA and proteins. Mice with colitis excreted bloody stools, lost weight, had shortened colon and underwent histopathological changes such as crypt loss and ulceration. Each animal was weighed in a random order. The test time was from 4:00 pm to 10:00 pm.

For fucoidan treatment, based on 7‐day 2.5% DSS induction, fucoidan (Aladdin, Cat# F304947, Shanghai, China) was injected (100 mg/kg) intraperitoneally daily for 7 days. In 10‐day colitis‐treatment models, fucoidan was injected (100 mg/kg) intraperitoneally daily from Day 4 to Day 10.

For EV treatment, based on 7‐day 2.5% DSS induction, 150 µg protein content of EVs from Caco‐2 cells (LPS‐EVs/CTRL‐EVs) were injected intraperitoneally on Days 0, 2, 4 and 6. For GW4869 treatment, based on 7‐day 2.5% DSS induction, GW4869 (2.5 mg/kg) was injected intraperitoneally on Days 0, 2, 4 and 6.

### 2.4 Blood Routine and Biochemical Examinations

Whole blood was collected in heparinized tubes, and then routine blood indexes, including the red blood cell count, haemoglobin concentration, and haematocrit, were determined by a Mindray automatic hematology analyzer (BC‐2800vet, Shenzhen, China).

For biochemical assays, blood was collected in heparinized tubes and centrifuged at 1200 × *g* for 15 min at 4°C. Plasma samples were prepared for determination of total iron binding capacity, unsaturated iron binding capacity, serum iron, and transferrin capacity by an auto‐chemical analyser (Beckman Coulter AU5421, Brea, CA, USA).

### 2.5 Histological Staining

For immunohistochemical assays, tissue sections were dewaxed for 10 min in xylene twice, hydrated for 5 min in each 100%, 95%, 85%, 70%, and 50% ethanol buffers sequentially, and rinsed for 5 min in running water at room temperature. After that, sections were treated in 3% H_2_O_2_ to inactivate catalase for 15 min and boiled in 10 mM sodium citrate (pH 6.0) to repair antigen for 10 min. Before being incubated with primary antibody overnight at 4°C, the tissue sections were blocked in 1% BSA for 1–2 h. After PBS washing three times and incubation with the secondary antibody from Jackson ImmunoResearch Laboratories for 3 h at room temperature, the tissue sections were put into a formulated mix containing 3,3’‐diaminobenzidin (DAB, ZSGB‐BIO, Beijing, China) for signal enhancement. The sections were then dehydrated and sealed with Permount Mounting Medium (Servicebio, Wuhan, China) for microscopic photography.

For immunofluorescent assays, the steps before the secondary antibody were the same as those for immunohistochemical assays. Slices were incubated with Alexa Fluor 488/594/647 conjugated secondary antibody. The nuclei were counterstained with 4’,6‐diamidino‐2‐phenylindole (DAPI) (Servicebio). Slides were drained and mounted using AntiFade Mounting Medium (Servicebio). Slides were visualized using a confocal microscope. Quantifications were performed with ImageJ software. For detailed information on primary antibodies, see Table S3.

For Prussian blue, Alcian blue and Periodic Acid‐Schiff (AB‐PAS), and haematoxylin and eosin (H&E) staining, the dewaxing steps were the same as those for histochemical assays. Slices were then incubated with the corresponding staining kit (Servicebio) following the manufacturer's instructions.

According to previously described methods (Niu et al. 2022), the histopathological scores of colon tissues were determined. Briefly, scoring rules are as follows: inflammatory cell infiltration (0: none, 1: mild, 2: moderate, 3: severe, 4: extremely severe), crypt injury (0: normal, 1: basal one‐third damaged, 2: basal two‐thirds damaged, 3: only surface epithelium intact, 4: entire crypt and epithelium lost), and extent of injury (0: none, 1: mucosal, 2: mucosal and submucosal, 3: transmucosal). The scores in each aspect were added to calculate a total score, with a higher score indicating a more severe lesion.

### 2.6 Quantitative Real‐Time PCR

Total RNA was isolated from colon tissue using the RNA isolater Total RNA Extraction Reagent (Vazyme Biotech, Nanjing, China) according to the manufacturer's specifications. Reverse transcription was used with the HiScript II 1st Strand cDNA Synthesis Kit (Vazyme Biotech). qPCR analysis was performed with ChamQ Universal SYBR qPCR Master Mix (Vazyme Biotech). *β‐actin* or *18S* was used as a control of the relative level of other genes. The relative mRNA expression levels were calculated using the delta‐delta CT method. The primers used are shown in Table S2.

### 2.7 Cell Culture

The human colonic epithelial cell line Caco‐2 cells (HTB‐37), mouse macrophage cell line RAW264.7 cells (TIB‐71) and human monocytic cell line THP‐1 cells (TIB‐202) were purchased from the American Type Culture Collection (ATCC, Rockville, USA). Caco‐2 and RAW264.7 cells were cultured in Dulbecco's modified Eagle medium (DMEM, Gibco, USA) supplemented with 10% foetal bovine serum (FBS, Gibco, USA) at standard conditions (37°C, 5% CO_2_). The THP‐1 cells were cultured in Roswell Park Memorial Institute‐1640 medium (RPMI‐1640, Gibco, USA) supplemented with 10% FBS under the same conditions. THP‐1 cells were incubated with 150 nM phorbol myristate acetate (PMA) for 24 h to differentiate into macrophages. For the co‐culture model, RAW264.7 cells were incubated in the lower compartment of 0.4 µm Transwell (Corning, Cat#3450, NY, USA), and Dendra2 or Dendra2‐FtH expressing Caco‐2 cells were incubated in the upper compartment.

The Malondialdehyde (MDA) levels of RAW264.7 cells were measured using the Lipid Peroxidation MDA Assay Kit (Beyotime, Jiangsu, China), which is based on the reaction of MDA and thiobarbituric acid (TBA). The absorptions were measured at 532 nm. MDA levels were calculated according to the established standard curve.

For RNA‐seq, RAW264.7 cells were divided into three groups and treated with PBS or EVs derived from Caco‐2 cells with or without LPS stimulation, corresponding to the CTRL, CTRL‐EV, and LPS‐EV groups, respectively. The cells were collected and rapidly frozen in liquid nitrogen and immediately sent to the commercial company (Tsingke, Beijing, China) for quality inspection and subsequent RNA sequencing.

### 2.8 Cell Transfection

The shRNA fragment targeting the *Msr1* gene, encoding macrophage scavenger receptor 1, was cloned into the lentiviral vector to construct the *Msr1* knockdown (KD) plasmid, transfected into RAW264.7 cells using a liposome transfection kit (Yeasen, Shanghai, China). After transfection, single clones were selected with 0.4 mg/mL puromycin. The total protein was extracted further to validate the KD efficiency by Western blot. The KD cells were also validated by Msr1 immunofluorescence assays. The obtained *Msr1* KD RAW264.7 cells were then used for further studies.

### 2.9 Flow Cytometry

After co‐culture, RAW264.7 cells were collected and resolved on BD FACS Calibur (BD Biosciences, NJ, USA) to detect Dendra2 fluorescent intensity. The levels of ROS of RAW264.7 cells with different treatments were assessed using a ROS assay kit (Yeasen), following the manufacturer's instructions. Briefly, the cell medium was removed and the cells were washed with PBS. Then the cells were incubated with the DCFH‐DA (10 µM) for 1 h at 37°C. After washing twice with serum‐free medium, the cells were collected, and the fluorescence intensity of the probe was examined using BD FACS Calibur (BD Biosciences).

### 2.10 EV Isolation and Characterization

Caco‐2 cells were cultured with a DMEM medium without 10% FBS until 70%–80% confluency. LPS (1 µg/mL, Sigma, Shanghai) and GW4869 (20 µM, Yeasen) were added into the medium. After an additional 24 h, culture supernatants of Caco‐2 cells were collected and then centrifuged at 300 × *g* for 10 min, 3000 × *g* for 20 min, and 10,000 × *g* for 30 min to remove the cells, dead cells, and cell debris, respectively. The EVs were isolated and purified by repeated ultracentrifugation of the supernatant twice at 100,000 × *g* for 70 min at 4°C. The size and concentration of EVs were evaluated using the NanoSight NS300 system (Malvern Panalytical, UK). A transmission electron microscope (HT7800, Hitachi, Tokyo, Japan) was used to observe the morphology and size of EVs.

For proteomics of EVs, CTRL‐EVs and LPS‐EVs were collected from Caco‐2 cells and immediately transported to the commercial company (Cosmos Wisdom, Hangzhou, China) on dry ice for quality inspection. Upon passing the quality check, a proteomics approach was performed.

### 2.11 Trichloroacetic Acid (TCA) Precipitation

The secretion assay was adapted from Hutchings AJ et al, with minor modifications (Hutchings et al. 2024). Briefly, 15 mL culture medium from Caco‐2 cells was collected and centrifuged firstly at 300 × *g* for 10 min, subsequently at 3000 × *g* for 20 min, at 10,000 × *g* for 30 min and lastly at 100,000 × *g* for 70 min. Then 2 mL of 100% TCA was added to the medium and mixed properly. Proteins were precipitated on ice at 4°C for 1 h and centrifuged at 16,000 × *g* for 30 min. The pellet was resuspended in protein lysis buffer for later usage.

### 2.12 Western Blots

Cells or tissues were harvested and washed with ice‐cold PBS and lysed in lysis buffer as previously described (Zhao et al. 2023). The protein concentrations of the lysate were measured using a standard Branford assay. For Western blots, 15–60 µg of protein from the whole cell lysate was electrophoresed by SDS‐PAGE. The proteins were transferred to the nitrocellulose membrane and probed with primary antibodies followed by horseradish peroxidase‐labelled secondary antibodies. The proteins were visualized using enhanced chemiluminescence and imaged by the digital Western blot imaging system (Tanon, Shanghai, China). Band intensity was quantified using ImageJ software.

### 2.13 Enzyme‐Linked Immunosorbent Assays (ELISA)

Cell medium supernatants and serum samples from mice were subjected to a sandwich ELISA using the human ferritin ELISA kit (Immunoway, Cat#KE1466) or mouse ferritin ELISA kit (Immunoway, Cat#KE1279) as per the manufacturer's instructions. Protein concentration corresponds to the O.D. value measured at 450 nm. Known concentrations of the protein (provided in the kit) were used to generate a standard curve to determine the concentration of ferritin in our samples.

### 2.14 Cy5‐Labelled FtH Construction

pETduet‐FtH plasmid was constructed. The His‐tagged FtH was expressed in *E. coli* (Arctic), induced by the addition of IPTG (0.2 mM). After cultivation for 16 h at 16°C, cells were collected by centrifugation. The pellets were resuspended in 50 mL of lysis buffer, sonicated on ice and centrifuged to remove *E. coli* debris. The supernatant was subjected to the Ni‐NTA column (Smart Lifesciences, Changzhou, China) to purify FtH.

Cy5‐labelled FtH was prepared with purified FtH proteins and Cy5‐NHS (Meilun, Dalian, China, Cat#146368‐14‐1), following the instruction manual. Briefly, the Cy5‐NHS ester was dissolved in DMSO of 1/10 reaction volume and the FtH proteins were dissolved in 0.1 M NaHCO3 solution of 9/10 reaction volume. Then both mixed. The mixture was vortexed constantly at room temperature for at least 4 h. To obtain the Cy5‐labelled FtH, the mixture was filtered using Amicon Ultra‐15 tubes.

### 2.15 Ferritin Iron Staining in Gels

CTRL‐EVs or LPS‐EVs were in SDS‐free sample buffers, followed by heating at 70°C for 10 min and centrifugation at 10,000 × *g* for 10 min. The supernatants were resolved on a native‐PAGE in non‐reducing conditions. The gel was stained with a Prussian Blue staining solution overnight at room temperature, then rinsed with distilled H_2_O until reaching a close‐to‐neutral pH and clear bands appeared.

### 2.16 Iron Content Detection

The intracellular Fe^2+^ of RAW264.7 cells after treatments were assessed using a FerroOrange fluorescent probe (F374, DOJINDO, Japan), following the manufacturer's instructions. Briefly, RAW264.7 cells were seeded in a laser confocal microscope dish, treated with FAC, Apo‐Ft, or Holo‐Ft for 24 h, and then the supernatant was discarded, and the cells were washed twice with PBS. These cells were incubated with 1 µmol/L FerroOrange at 37°C for 30 min in a cell incubator. Subsequently, the supernatant was discarded, and the cells were washed twice using PBS. The cells were then examined using a confocal fluorescence microscope FV3000 (Olympus, Tokyo, Japan). The intracellular fluorescence intensity of the probe was quantified using the ImageJ software.

The iron contents were also assessed using a Ferrozine chromogenic method. Briefly, protein‐binding iron is dissociated by hydrochloric acid and 100°C heating for 15 min. Dissociated iron is then reduced by vitamin C and forms a chelate with the Ferrozine chromogen. The intensity of this coloured complex is proportional to the iron concentration in the sample and is measured at 570 nm.

The levels of the labile iron pool (LIP) were detected using a Calcein method (Li et al. 2019). Briefly, 1 × 10^6^ RAW264.7 cells were collected and resuspended with PBS. These cells were incubated with 125 nM Calcein‐AM for 15 min at 37°C. After washing twice with PBS, the cell suspension was added to a 96‐well plate, and fluorescent intensity was assessed at 488 nm by microplate reader SpectraMax M3 (Molecular Devices, SJ, USA). Then, DFP was added to the cell suspension to reach 100 µM. After shaking the plate softly, the fluorescent intensity was reread within 5 min. The increment of fluorescence indicated the levels of LIP.

### 2.17 Statistical Analysis

All data were presented as the mean ± SEM. Statistical analyses were conducted using Student's *t*‐test between 2 groups, while ANOVA was used between 3 or more groups. GraphPad Prism 9.0 (GraphPad Software, USA) was used to perform statistical analyses. *p* < 0.05 indicates statistical significance.

## 3 Results

### 3.1 Iron Deposits in Macrophage‐Rich Intestinal Lamina Propria in IBD Patients and Mice With DSS‐Induced Colitis

**FIGURE 1:**
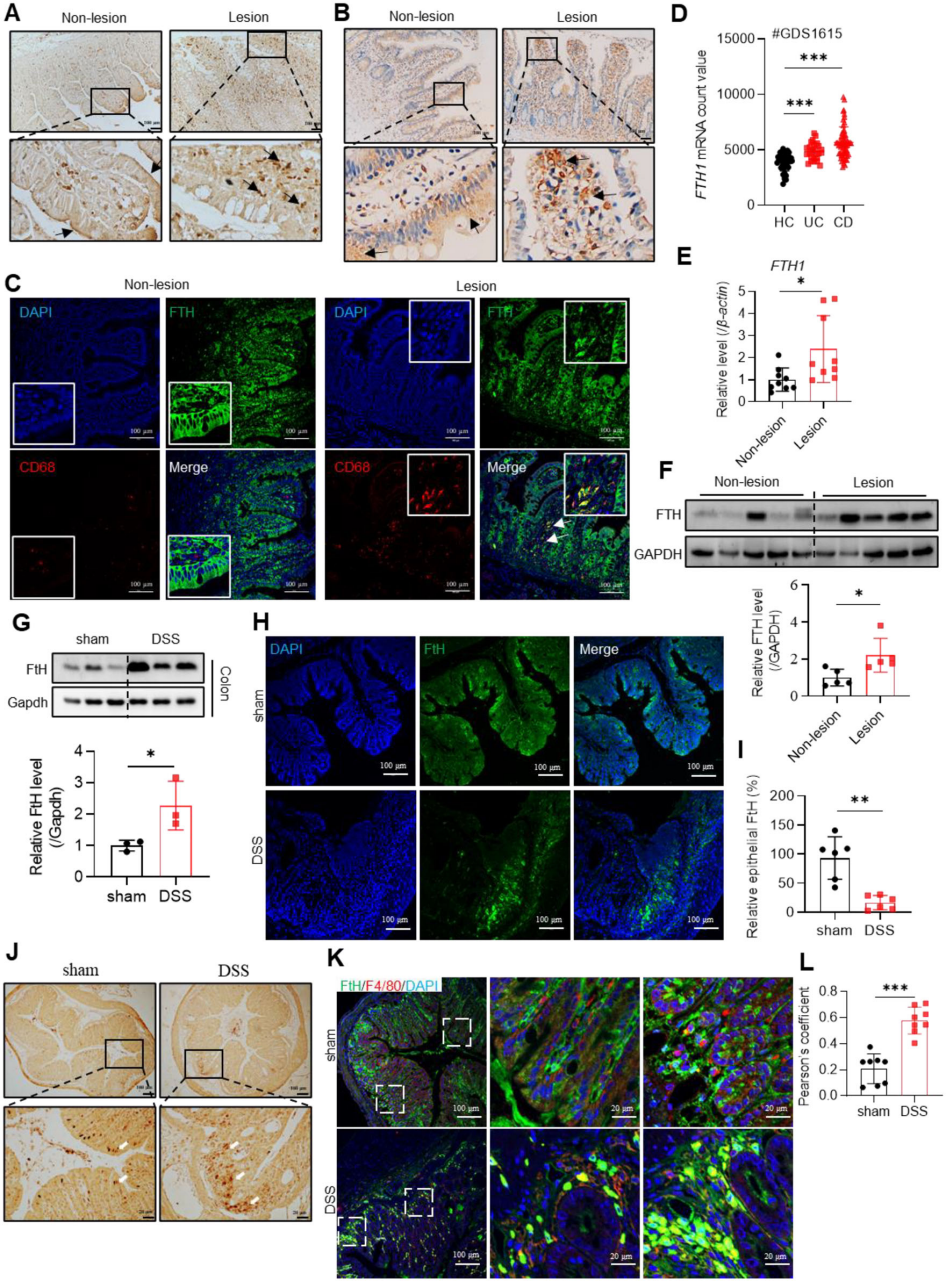
The expression patterns of FTH are changed in IBD patients and DSS‐induced murine colitis model, in which FtH decreases in epithelial cells and increases in macrophages at protein levels. (A) Representative images of DAB‐enhanced Prussian blue iron‐stained intestinal sections from IBD patients. Arrows indicate iron deposit. *N* = 10/group. Bar: 100 µm. (B) Representative images of immunohistochemistry for FTH in intestinal sections from IBD patients. Arrows indicate FTH positive area. Bar: 100 µm. (C) Confocal images of the FTH (green) and macrophage marker CD68 (red) in intestinal sections from IBD patients. Arrows indicate co‐localization. Nuclei were labelled with DAPI (blue). Bar: 100 µm. (D) *FTH1* mRNA levels in peripheral blood mononuclear cells from IBD patients (UC: ulcerative colitis; CD: Crohn's disease). The data were collected from GEO profile GDS1615. (E) *FTH1* mRNA levels in intestinal sections from IBD patients collected in this study, revealed by RT‐qPCR. *N* = 9/group. (F) FTH protein levels in intestinal sections from IBD patients, revealed by western blotting. *N* = 5/group. (G–L) Results from 2.5% DSS‐induced murine colitis model. (G) FtH protein levels in colon tissue and quantification of the band intensity. (H) Confocal images of FtH in colon tissues. (I) Quantification of fluorescence intensity for epithelial FtH. (J) DAB‐enhanced Prussian blue iron‐stained colon tissues. (K) Confocal images of FtH and macrophage marker F4/80. (L) Pearson's coefficient for macrophage (F4/80 in red) and FtH (in green) overlay. Nuclei were labelled with DAPI (blue). Bar: 20 µm. Values are shown as mean ± SEM. *t*‐test was used for the comparison between the two groups. One‐way ANOVA was utilized for the comparison among more than 3 groups. *p < 0.05, **p < 0.01, ***p < 0.001.

In previous work, we found inflammation‐mediated modifications of iron distribution in IBD patients and mice (Harrer et al. 2025). We further validated these observations in the intestinal lesion areas of IBD patients (Table S1). Specifically, iron is more abundant in the lamina propria than in the IECs in the lesion sections, whereas the opposite is true in the normal sections (Figure 1A). Notably, the FTH subunit was predominantly detected in the lamina propria of intestinal lesions from IBD patients, while it was primarily localized in epithelial cells in controls, as shown by immunohistochemical assays (Figure 1B) and immunofluorescent assays (Figure 1C). The lamina propria contains a diverse array of immune cells, including tissue‐resident and/or myeloid macrophages, which are at the frontline in the defence against pathogen infection. The increased ferritin in lesion regions was found in macrophages, revealed by a significant co‐localization of FTH with macrophage marker CD68 (Figure 1C). Consistently, *FTH1* mRNA levels were increased in the peripheral blood mononuclear cells of IBD patients, as extracted from the GEO database (#GDS1615) (Figure 1D), and in the intestinal samples collected from the IBD patients in this study (Figure 1E), in line with protein levels (Figure 1F). Since ferritin is primarily regulated post‐transcriptionally under normal conditions, these mRNA data suggest that inflammation substantially contributes to the expression of *FTH1* in IBD patients (Pham et al. 2004).

To confirm the iron distribution in an IBD mouse model, we treated mice with DSS and measured the length of the colon, body weight, and inflammatory cytokines and performed H&E staining (Figure S1A–G). Consistent with previous studies (Xu et al. 2020; Moon et al. 2022), systemic iron homeostasis was disrupted (Figure S1H–O). Similar to human observations, total FtH was elevated in the colon of mice with DSS‐induced colitis (Figure 1G). However, FtH levels were lower in the epithelial layer (Figure 1H, quantified in 1I), while increased iron (Figure 1J) and greater co‐localization of macrophage marker F4/80 and FtH (Figure 1K,L) were observed in lamina propria. Therefore, the localization patterns of FtH and iron deposition shift in the context of IBD in both human patients and a mouse model of the disease.

### 3.2 *FtH* Depletion in IECs, Not in Macrophages, Alleviates the Severity of DSS‐Induced Colitis in Mice

Since the intestinal lamina propria is primarily composed of various cell types, including different immune cells, fibroblasts, vascular endothelial cells, smooth muscle cells, adipocytes, and neurons, we asked whether the FTH changes under IBD conditions are related to these cell types. Co‐localization of FtH with cell type markers was examined under both non‐inflammatory and DSS‐induced inflammatory conditions in the mouse colon tissue using immunofluorescence (Figure S2A–H). The results showed that FtH exhibited low levels in enteric neurons (MAP2), smooth muscle cells (α‐SMA), and fibroblasts (Vimentin) and not‐detectable levels in vascular endothelial cells (CD31), dendritic cells (CD11c), adipocytes (FABP4), and lymphocytes (CD3/CD19) with no significant difference before and after DSS induction. These results strongly suggest the critical roles of FTH in macrophages.

**FIGURE 2:**
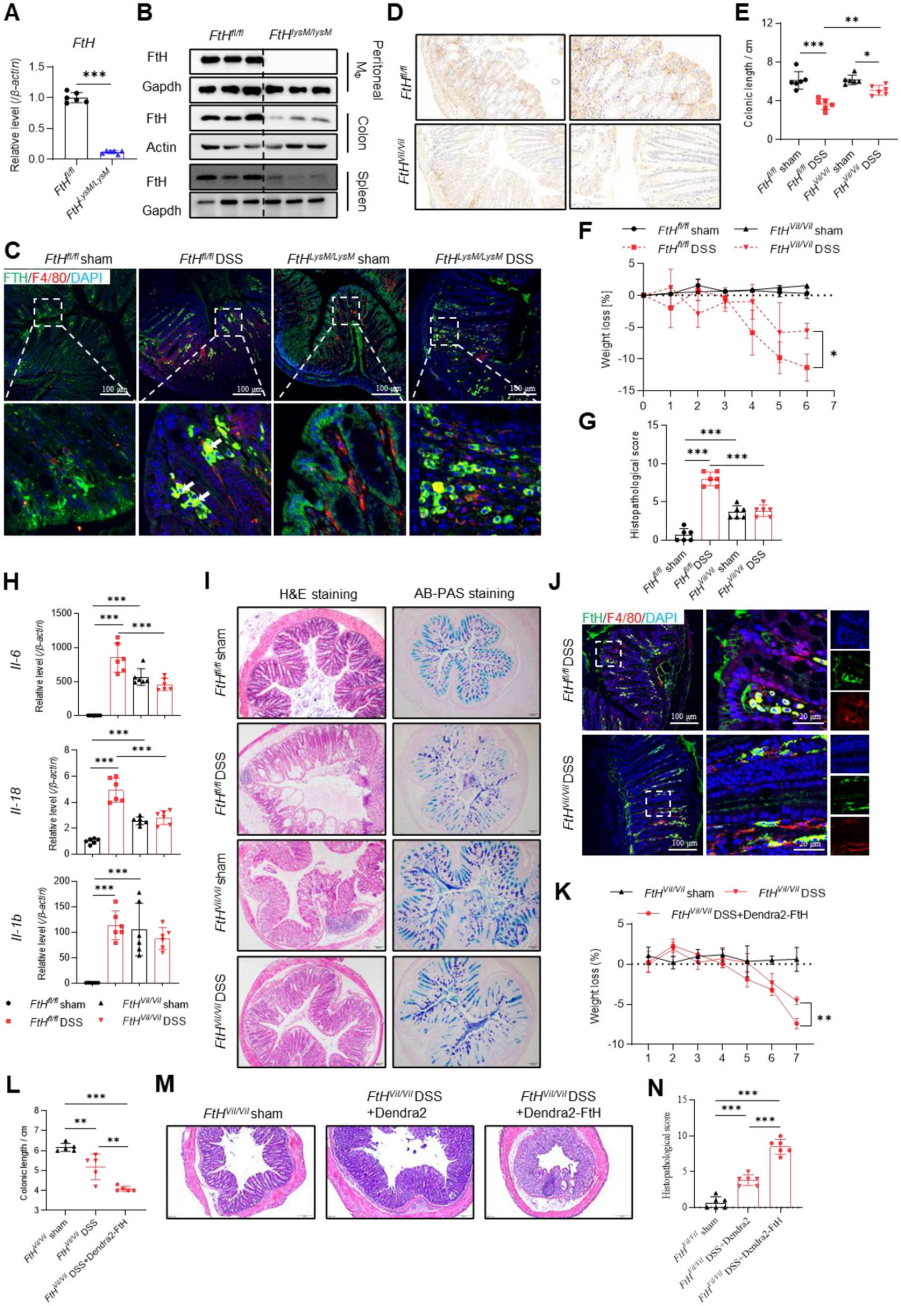
*FtH* depletion in IECs, not in macrophages, alleviates the severity of DSS‐induced colitis in mice. (A) *FtH* mRNA levels in bone‐marrow‐derived macrophages (BMDM). (B) Protein levels of FtH in peritoneal macrophages and macrophage‐rich tissues (colon, spleen) revealed by western blotting. (C) Confocal images showing FtH (green) and F4/80 (red). Nuclei were labelled with DAPI (blue). Bar: 100 µm. (D) Representative images of anti‐FtH immunohistochemistry in intestinal sections from *FtH^fl/fl^* and *FtH^Vil/Vil^* mice. Bar: 100 µm. (E) Quantification of colonic length. (F) Body weight loss. (G) Histopathological scores. (H) mRNA levels of inflammatory genes of colon tissue detected by RT‐qPCR. (I) Hematoxylin and eosin (H&E)‐stained and Alcian Blue‐Periodic Acid Schiff (AB‐PAS)‐stained colon tissues. Bar: 100 µm. (J) Representative images of immunofluorescence for FtH (green) and F4/80 (red). Quantification of percentage of FtH^+^F4/80^+^ cells. Nuclei were labelled with DAPI (blue). Bar: 100 µm. (K–N) Dendra2‐FtH‐expression via plasmid/PEI in IECs of *FtH^Vil/Vil^* mice. Post‐72 h, the mice drank 2.5% DSS water for 7 days. Bar: 30 µm. (K) Weight loss. (L) Colon length. (M) Histology of colon section, revealed by H&E staining. Bar: 100 µm. (N) Histopathological scores. Values are shown as mean ± SEM. t‐test was used for the comparison between the two groups; one‐way ANOVA was utilized for the comparison among more than 3 groups and two‐way ANOVA was utilized for the body weight loss. *N* = 6. *p < 0.05, **p < 0.01, ***p < 0.001. PEI: polyethyleneimine.

To investigate the role of macrophage *FtH* in the development of DSS‐induced colitis, we generated myeloid‐specific *FtH* knockout mice (*FtH^LysM/LysM^*). This was confirmed by the absence of *FtH* mRNA in bone marrow‐derived macrophages (BMDMs) (Figure 2A) and protein in peritoneal macrophages (Figure 2B) from *FtH^LysM/LysM^* mice. The protein levels of FtH were also reduced in macrophage‐rich colon and spleen tissues (Figure 2B). Surprisingly, the severity of DSS‐induced colitis in *FtH^LysM/LysM^* mice was similar to that in *FtH^fl/fl^* mice across all tested parameters (Figure S3A–J). We evaluated the impact of *FtH* deficiency on the inflammatory response and phagocytic function of macrophages. The RT‐qPCR results demonstrated that BMDMs with *FtH* deficiency exhibited a reduced inflammatory response to LPS stimulation (Figure S3K). Meanwhile, there was an increased expression of receptor genes involved in the phagocytosis of heme and HGB (Figure S3L). Following the phagocytosing of oxidized low‐density lipoprotein (ox‐LDL), oil red staining revealed a significant increase in lipid accumulation (Figure S3M). These results suggested that the phagocytotic function of *FtH* deficient BMDM was enhanced. However, FtH was still detectable in macrophages around lesion areas and was colocalized with F4/80 after DSS treatment in *FtH^LysM/LysM^* mice (Figure 2C). This led us to question whether ferritin is derived from FtH‐rich IECs under inflammatory conditions.

To address the contribution of IECs FtH in the pathophysiology of IBD, we generated IECs‐specific *FtH*‐depleted mice (*FtH^Vil/Vil^*) by crossing the *FtH^fl/fl^* with *Vil^Cre/Cre^* mice. Immunohistochemical staining confirmed the depletion efficiency (Figure 2D). Consistent with previous results (Vanoaica et al. 2010), *FtH^Vil/Vil^* mice showed decreased intestinal and increased systemic iron content (Figure S4A,B). No significant differences were observed in terms of colonic length and body weight, but histopathological scores, between *FtH^fl/fl^* and *FtH^Vil/Vil^* mice before DSS treatment (Figure 2E–G). However, the difference was remarkable after DSS treatment (Figure 2E–G). Consistently, the expression levels of inflammatory genes in the colon were upregulated in *FtH^Vil/Vil^* mice compared to *FtH^fl/fl^* before DSS treatment, as previously reported (Vanoaica et al. 2010). However, these levels did not further elevate in *FtH^Vil/Vil^* mice after DSS treatment, with a much lower inflammatory response to DSS treatment in *FtH^Vil/Vil^* mice than in *FtH^fl/fl^* mice (Figure 2H). Similar results were revealed by subsequent histological analysis, characterized by reduced epithelial disruption, crypt loss, and erosion in *FtH^Vil/Vil^* mice compared to *FtH^fl/fl^* mice (Figure 2I, left panel), and mucin staining, by a slight variation in dark and light blue in *FtH^Vil/Vil^* mice before and after colitis establishment (Figure 2I, right panel). These results indicate that IECs FtH is involved in the pathophysiology of IBD.

Then we detected co‐localization between FtH and F4/80 and found much less co‐localization in colon of *FtH^Vil/Vil^* mice than in of *FtH^fl/fl^* mice after DSS treatment (Figure 2J). By comparing the expression levels of FtH in inflammatory colons with immunoblotting, *FtH^Vil/Vil^* mice presented decreased levels of FtH, while *FtH^LysM/LysM^* mice did not (Figure S4C), suggesting the large contribution of villi to colonic FtH. When Dendra2‐labelled FtH was overexpressed in IECs of *FtH^Vil/Vil^* mice, mediated by *in vivo* PEI clyster (Huang et al. 2014; Sun et al. 2019), we found the exacerbation of disease severity, evidenced by increased weight loss (Figure 2K), reduced colonic length (Figure 2L), and worsened histology (Figure 2M) and histopathological score (Figure 2N), indicating that IECs FtH contributes to DSS‐induced inflammation in mice.

### 3.3 DSS‐Induced Intestinal Inflammation Elicits Ferritin Trafficking From IECs to Macrophages via EVs

**FIGURE 3:**
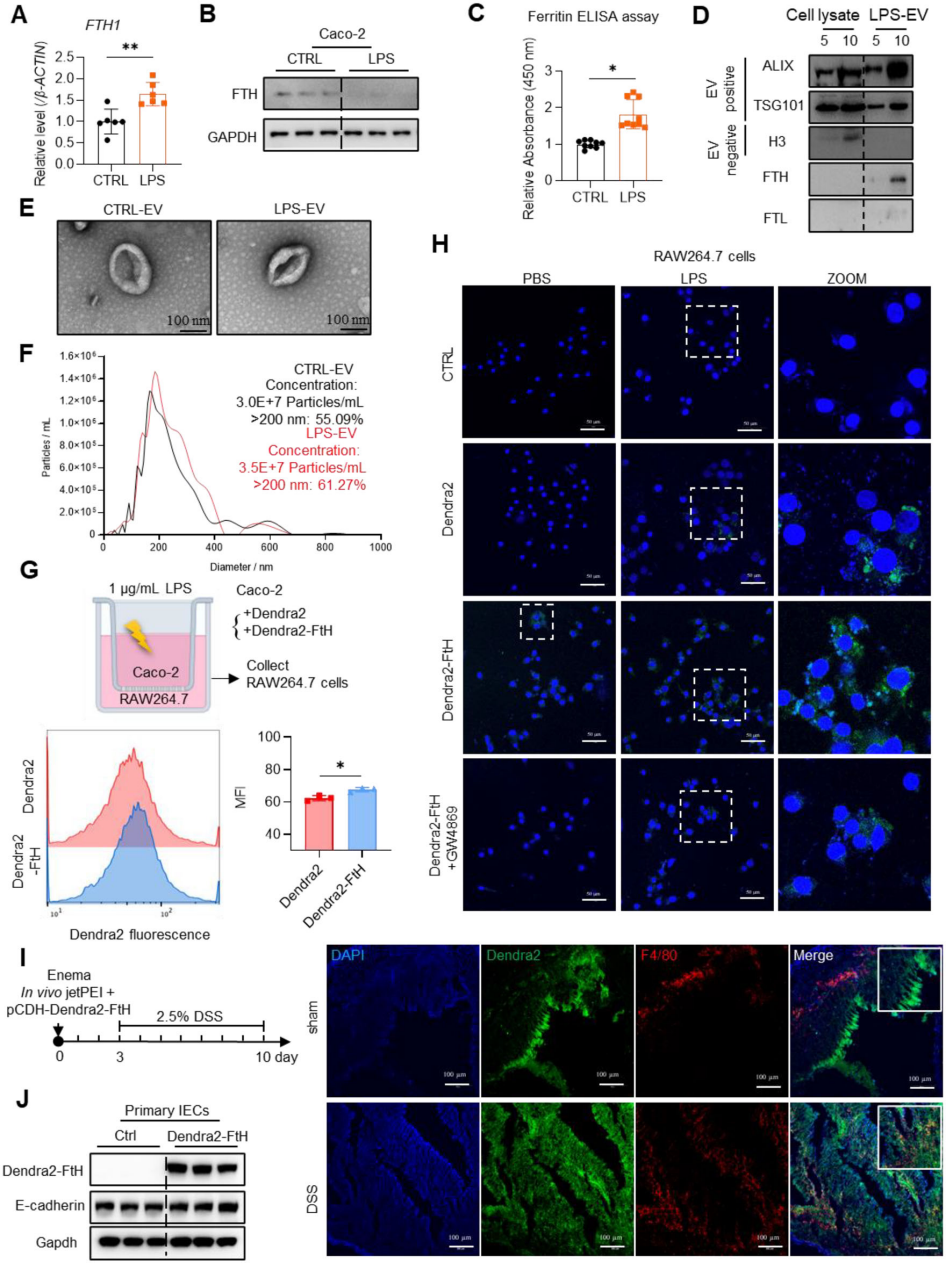
DSS‐induced intestinal inflammation elicits ferritin trafficking from IECs to macrophages via EVs. A‐C: In lipopolysaccharide (LPS)‐treated intestinal epithelial cell line Caco‐2. (A) mRNA levels of *FTH1* detected by RT‐qPCR. (B) Protein levels of FTH detected by western blotting. (C) Secreted ferritin in culture medium, detected by ELISA assay. (D) Protein analysis of EVs purified by ultracentrifugation from culture medium of Caco‐2 cells after LPS stimulation for 24 h. EVs were resuspended with 100 µL PBS. Cell lysates (5/10 µg proteins) and EVs (5/10 µL PBS solution) were blotted for FTH, the exosomal positive markers ALIX and TSG101, and exosomal negative marker H3. (E) Representative electron microscopy images of EVs. Bar: 100 nm. (F) Diameter of EVs analysed by nanoparticle tracking system. (G) Scheme of Transwell and Dendra2 mean fluorescent intensity (MFI) detected by flow cytometry. Caco‐2 (Dendra2 or Dendra2‐FtH expression) cells were incubated in the upper chamber while RAW264.7 cells were seeded in the lower well. After LPS induction for 24 h, RAW264.7 cells were collected. (H) Representative images of Dendra2 fluorescence in RAW264.7 cells, which were co‐cultured with Caco‐2 cells expressing Dendra2 or Dendra2‐FtH, pretreated with GW4869 and/or LPS as indicated for 24 h. CTRL group means RAW264.7 cells only without co‐culture. Bar: 50 µm. (I) Diagram of the in vivo experimental design. (J) Successful delivery of Dendra2‐FtH‐expressing plasmid through in vivo jetPEI to mouse IECs and FtH trafficking from IECs to macrophages detected by western blotting and immunofluorescence, respectively. Macrophages were labelled with F4/80 (red); nuclei were labelled with DAPI (blue). Bar: 100 µm. Values are shown as mean ± SEM. *t*‐test was used for the comparison between the two groups. One‐way ANOVA was utilized for the comparison among more than 3 groups. Lipopolysaccharide (LPS, 1 µg/mL) was used to treat cells for 24 h. *p < 0.05, **p < 0.01, ***p < 0.001.

To test a hypothesis that ferritin traffics from epithelial cells to macrophages to raise inflammation, we treated epithelium‐like Caco‐2 cells with lipopolysaccharide (LPS). We found increased mRNA levels of *FTH1* (Figure 3A), but intracellular protein levels of FTH were reduced (Figure 3B) and extracellular FTH increased in the culture medium (Figure 3C). In contrast, both *FtH* mRNA and intracellular protein levels increased in macrophage‐like RAW264.7 cells after LPS treatment without more extracellular FtH (Figure S5A–C). Previous studies, including ours, have demonstrated ferritin secretion via EVs (Truman‐Rosentsvit et al. 2018; Brown et al. 2019; Chen et al. 2023). To determine if IECs secreted ferritin via EVs in an inflammatory context, we isolated EVs from the culture medium of Caco‐2 cells by ultracentrifugation and characterized them by immunoblotting, transmission electron microscopy, and nanoparticle tracking analysis. Consistent with our hypothesis, FTH was enriched in the EVs (Figure 3D) without morphological changes (Figure 3E). The particle counts increased, and the size pattern was right‐shifted in LPS‐EVs (Figure 3F). To determine what percentage of the secreted FTH is in EV‐coated form or free form in the medium, we precipitated the supernatants post ultra‐centrifugation using the TCA (Figure S6A) and detected FTH levels by Western blotting. The secreted ferritin was primarily enriched in EV fraction, even though the total protein in TCA precipitated fraction was greater (Figure S6B). These results indicated that most of the secreted FtH protein from Caco‐2 cells is encapsulated within EVs, rather than being in a free form.

To provide more solid evidence for FTH contribution, we conducted proteomic analysis on CTRL‐EVs and LPS‐EVs. The results showed that the protein composition differences between the two groups were quite pronounced (Figure S6C). Notably, FTH was the second most upregulated in LPS‐EVs after a RhoGTPase‐activating protein 17 (ARHGAP17) (Figure S6D). Based on Arhgap17's protective function in DSS‐induced colitis (Lee et al. 2016), we propose that EV‐coated ferritin plays a prominent role in modulating the immune response. The roles of other increased or decreased proteins need intensive study. More omics analysis (Figure S6E–I) indicated that EV protein compositions are involved in cellular metabolic processes, binding, immune system, and signal transduction.

To further elucidate the fate of secreted ferritin, we employed the Caco‐2/RAW264.7 co‐culture model to simulate the intestinal microenvironment (Figure 3G, top panel). Caco‐2 cells were engineered to stably overexpressed Dendra2 or Dendra2‐fused FtH (Dendra2/Dendra2‐FtH). Post‐LPS stimulation, an increase in fluorescent density within RAW264.7 cells was observed via flow cytometry (Figure 3G) and direct fluorescence detection following co‐culture (Figure 3H). The fluorescent signal from Dendra2‐FtH‐overexpressing Caco‐2 cells was already sporadically detectable in RAW264.7 cells prior to LPS stimulation (Figure 3H). However, this signal was significantly enhanced by LPS‐EVs and diminished when EV secretion was inhibited using GW4869 (Figure 3H). As demonstrated by fluorescence assays (Figure S7A) and flow cytometry (Figure S7B), the EVs, isolated from Caco‐2 cells and labelled with fluorescent dyes (PKH67 or PKH26), were directly internalized by RAW264.7 cells. The internalization was further enhanced by LPS‐stimulated Caco‐2‐derived EVs. These in vitro findings support the hypothesis of enhanced ferritin trafficking from epithelial cells to macrophages via the EV pathway in the context of inflammation. To further validate the hypothesis in vivo, we utilized PEI‐mediated clyster to express Dendra2‐FtH in IECs (Figure 3I,J). E‐cadherin served as a marker for IECs. We observed the colocalization of Dendra2‐FtH (green) and macrophages (red) only in DSS‐treatment group (Figure 3J, right images). This finding corroborates the transfer of FtH from IECs to macrophages, induced by intestinal inflammation.

**FIGURE 4:**
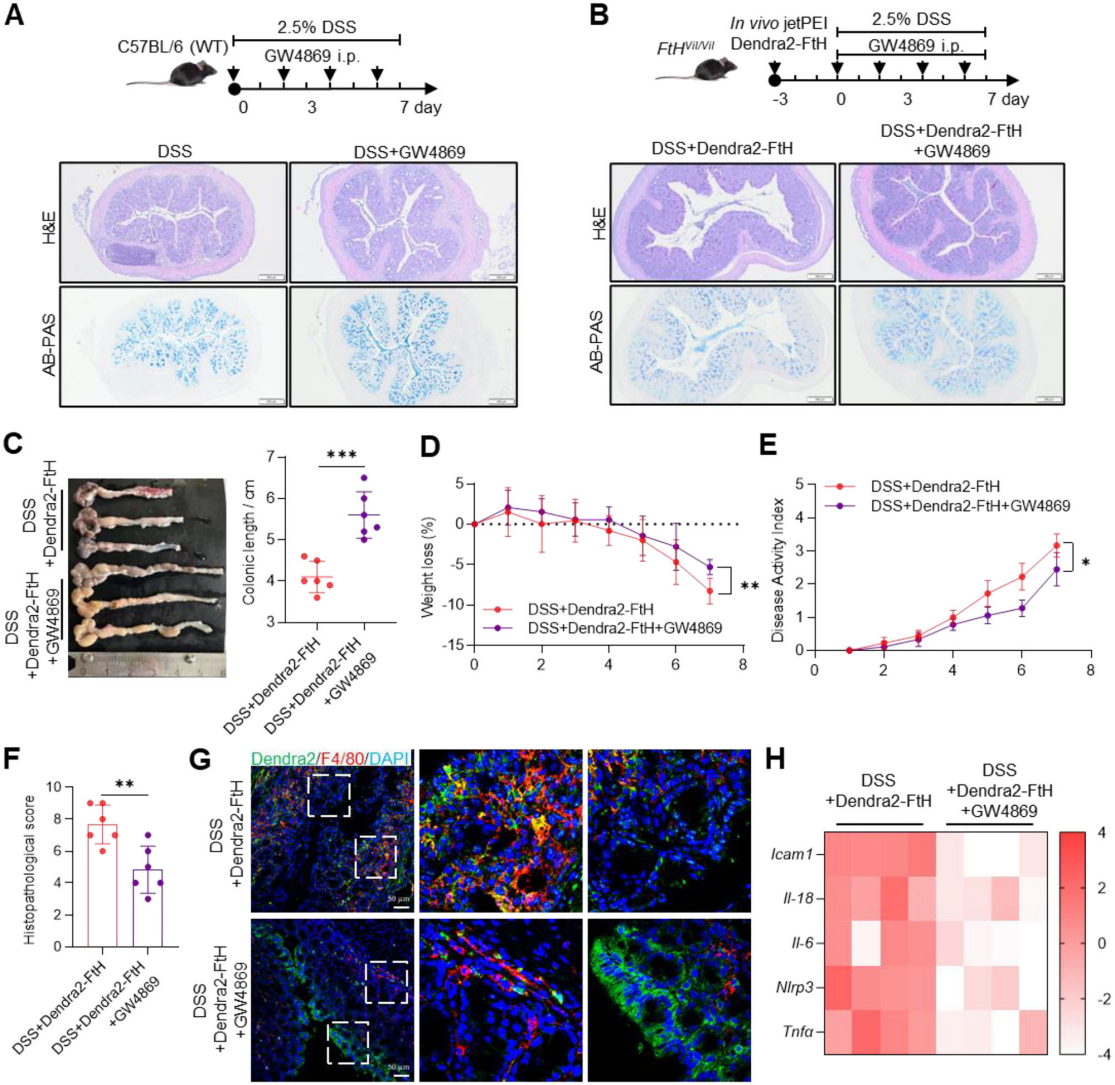
EV inhibitor GW4869 reduces ferritin transfer from IECs to macrophages. (A) C57BL/6 (WT) mice were given 2.5% DSS for 7 days and intraperitoneally injected with GW4869 in Days 0, 2, 4, 6. Experimental design schematic diagram and H&E and AB‐PAS staining of colon tissues. Bar: 200 µm. (B) *FtH^Vil/Vil^* mice were overexpressed Dendra2‐FtH using in vivo jetPEI and given 2.5% DSS for 7 days. These mice were intraperitoneally injected with GW4869 in Days 0, 2, 4, 6. Experimental design schematic diagram and H&E and AB‐PAS staining of colon tissues. Bar: 200 µm. (C) Representative image of colon tissues and quantification of colonic length. (D) Weight loss. (E) Disease activity index. (F) Histopathological score. (G) Confocal images of the Dendra2‐FtH (green) and macrophage marker F4/80 (red) in colon tissues. Nuclei were labelled with DAPI (blue). Bar: 50 µm. (H) Heatmap of inflammatory genes expression levels detected by RT‐qPCR. Values are shown as mean ± SEM. *t*‐test was used for the comparison between the two groups. *p < 0.05, **p < 0.01, ***p < 0.001.

To reveal the role of EV‐mediated communication between IECs and macrophages in the process of DSS‐induced colitis, we used the EV inhibitor GW4869 to interfere with EV release in WT and *FtH^Vil/Vil^* mice overexpressing FTH in the epithelium via in vivo jetPEI (Figure 4A,B). The colon histology, revealed by H&E staining, and secreted mucus by AB‐PAS staining both indicated the improvement after EV‐release inhibition (Figure 4A,B). Other phenotypes were also significantly improved, including colonic length (Figure 4C), weight loss (Figure 4D), disease activity index (Figure 4E), and histopathological score (Figure 4F). Notably, the Dendra2‐FtH (green) and macrophage marker F4/80 (red) showed strong co‐localization in inflamed colon, whereas GW4869 significantly blocked Dendra2‐FtH relocation from IECs to macrophages (Figure 4G). Reduced inflammatory response was further confirmed by inflammatory gene expression (Figure 4H). These results indicate that EV‐mediated FtH trafficking participates in the development of IBD.

### 3.4 Iron‐Loaded Ferritin‐Containing EVs Promote Oxidative Stress and Inflammation in Macrophages

**FIGURE 5:**
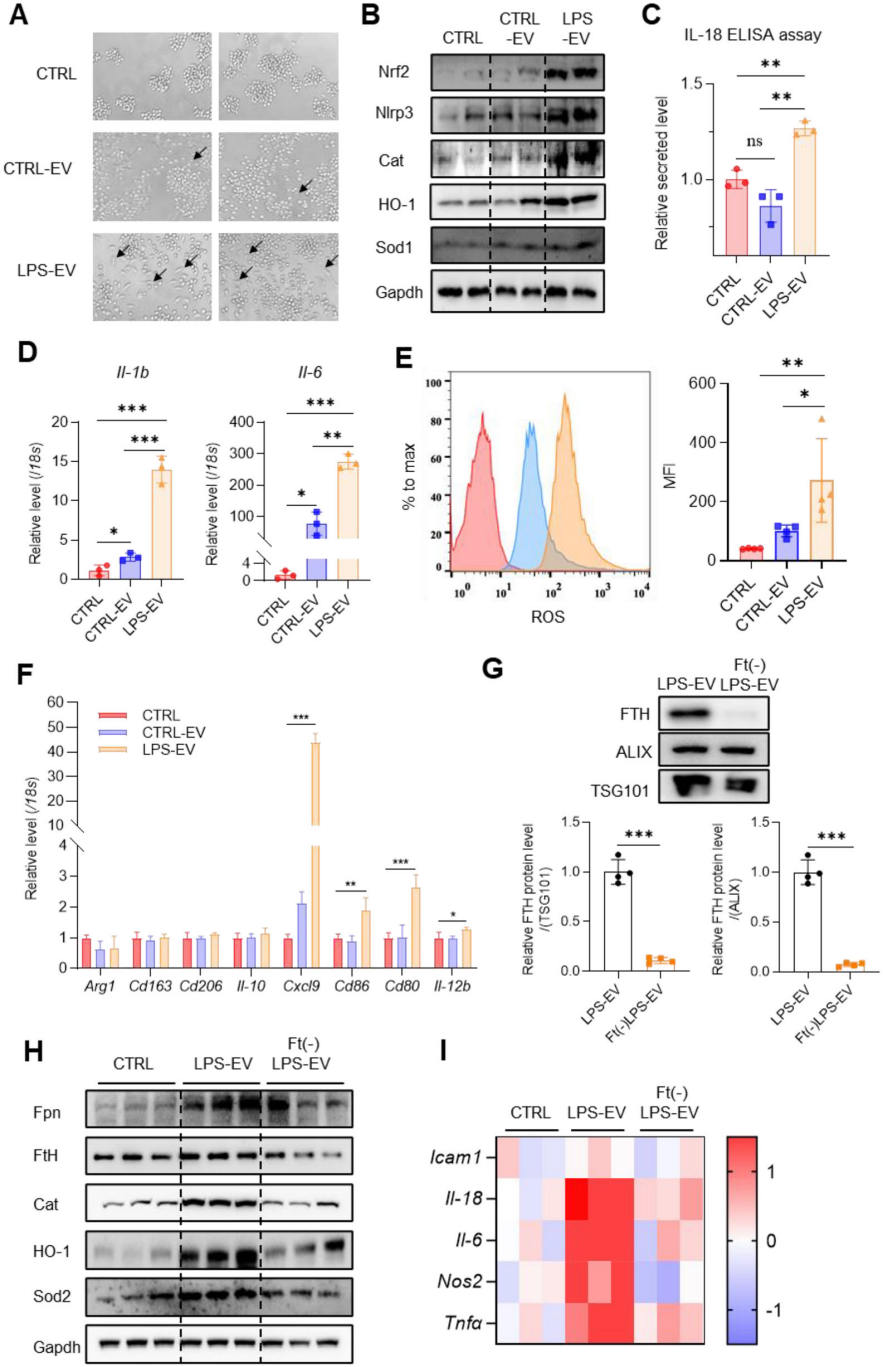
EVs from IECs trigger inflammation and oxidative stress in macrophages. Caco‐2 cells were stimulated with/without LPS and their EVs were collected (CTRL‐EV/LPS‐EV). RAW264.7 cells were treated with the EVs. (A) Cell morphology observed by microscope. (B) Levels of inflammation‐ and oxidative stress‐associated proteins, detected by western blotting. (C) Secreted IL‐18 detected by ELISA assays. (D) mRNA levels of *Il‐1b* and *Il‐6* detected by RT‐qPCR. (E) ROS levels indicated by DCFH‐DA using flow cytometry and the quantification of mean fluorescence intensity. (F) The expression levels of M1/M2 phenotype markers detected by RT‐qPCR. (G–I) RAW264.7 cells were treated with same quantity of EVs from LPS‐stimulated Caco‐2 cells with/without removal of FtH (LPS‐EV/Ft(‐)LPS‐EV). (G) The protein levels of FTH in LPS‐EVs and Ft(‐)LPS‐EVs and corresponding quantification. (H) Levels of inflammation‐ and oxidative stress‐associated proteins in RAW264.7 cells detected by western blotting. (I) Heatmap for relative mRNA levels of inflammatory genes in RAW264.7 cells detected by RT‐qPCR. Values are shown as mean ± SEM. *t*‐test was used for the comparison between the two groups. One‐way ANOVA was utilized for the comparison among 3 groups. *p < 0.05, **p < 0.01, ***p < 0.001.

To further investigate the impacts of EV‐coated FtH from IECs to macrophages under inflammatory conditions, we isolated EVs from Caco‐2 cells pretreated with LPS (LPS‐EVs) to treat RAW264.7 cells. The cell morphology underwent a significant alteration with long pseudopodia by adding LPS‐EVs (Figure 5A). The expression of inflammation and oxidative stress‐associated proteins were upregulated after LPS‐EV treatment, detected by immunoblotting (Figure 5B for Nrf2, Nlrp3, catalase (Cat), heme oxygenase 1 (HO‐1), and superoxide dismutase 1 (Sod1)), ELISA (Figure 5C for IL‐18), and RT‐qPCR (Figure 5D for *Il‐1b* and *Il‐6*). The levels of reactive oxidative species (ROS), indicated by DCFH‐DA using flow cytometry, were also increased in LPS‐EV treated RAW264.7 cells (Figure 5E), indicating low levels of inflammatory response when treated with CTRL‐EVs, but boosted‐up response when treated with LPS‐EVs. To study the effects of these EVs on gene expression profiling of CTRL‐EV or LPS‐EV‐stimulated RAW264.7 cells, we performed RNA‐sequencing (Figure S8A,B). Consistently, we observed an upregulation of oxidative stress genes, inflammatory cytokines & chemokines, and MAPK signalling pathway in macrophages treated with LPS‐EVs (Figure S8C–G). Glycolysis‐related genes were also upregulated, while oxidative phosphorylation‐related genes were downregulated (Figure S8H), all of which suggest a shift of macrophages towards a pro‐inflammatory phenotype. We validated the transcriptomic results by detecting genes associated with macrophage polarization by RT‐qPCR. Expression of pro‐inflammatory genes (*Cxcl9, Cd86, Cd80, Il‐12b*) in macrophages significantly increased after LPS‐EV treatment, while Expression of anti‐inflammatory genes (*Arg1, Cd163, Cd206, Il‐10*) showed no significant changes (Figure 5F). We repeated the experiments with THP‐1 cells, a human monocytic cell line, differentiated into macrophages by PMA induction, and obtained similar results (Figure S9A). To further validate the role of FtH in promoting inflammation and oxidative stress, we altered the EV membrane permeability by sonication for release of the EV‐packed ferritin (Zhao et al. 2021). An FtH‐antibody‐coated ELISA plate was utilized to remove ferritin to generate ferritin‐depleted EVs (Ft(‐)LPS‐EVs) (Figure 5G). Then RAW264.7 cells were treated with the Ft(‐)LPS‐EVs, and inflammatory and oxidative stress‐associated molecules were examined. We found, though the same quantity of EVs, that the observed effects vanished in Ft(‐)LPS‐EV‐treated cells, detected by immunoblotting (Figure 5H) and qPCR (Figure 5I).

**FIGURE 6:**
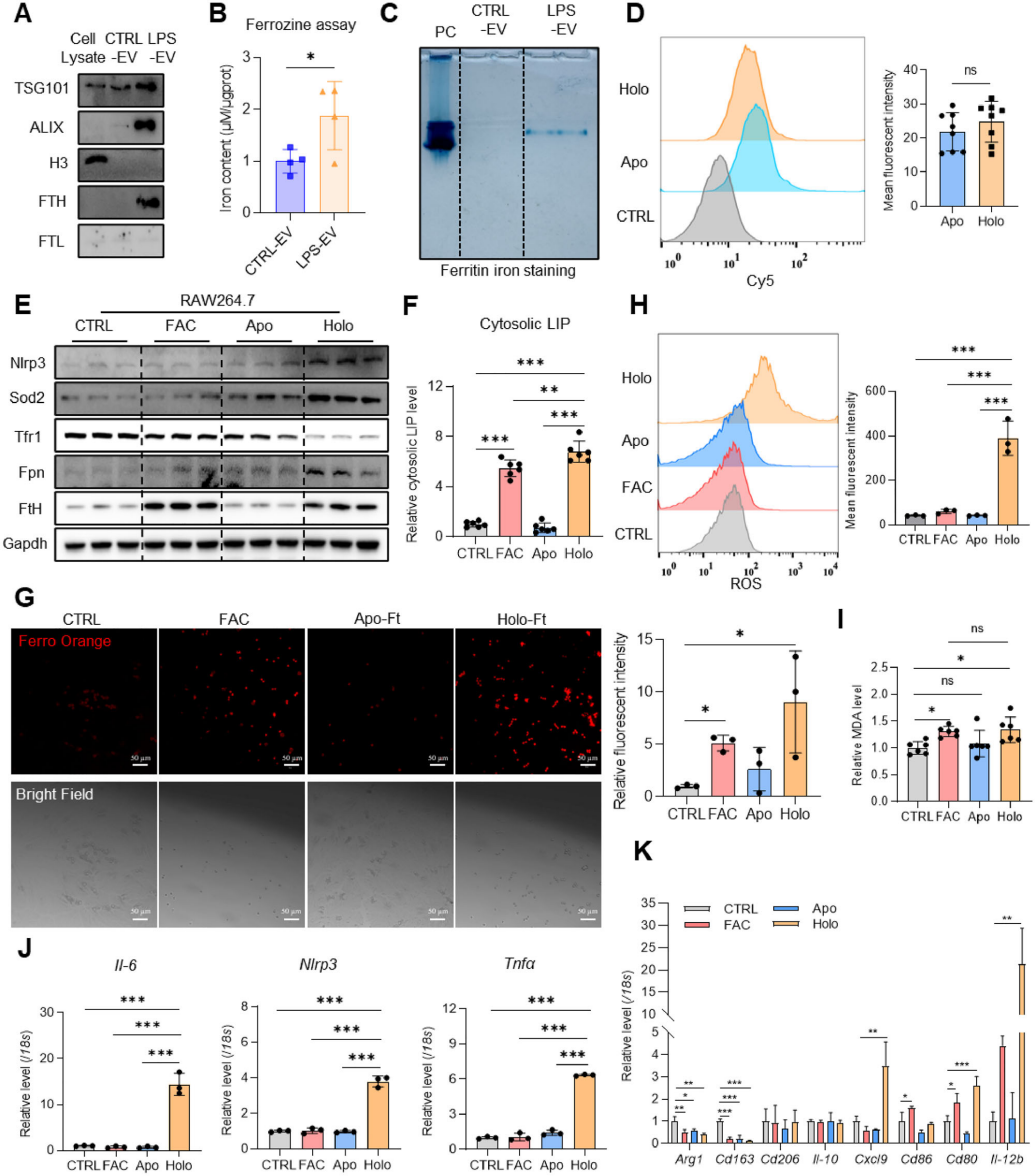
Uptake of iron‐loaded ferritin promotes oxidative stress and inflammation in macrophages. (A) Protein levels of FTH in EVs from Caco‐2 with/without LPS stimulation detected by western blotting. (B) Iron contents of EVs from Caco‐2 with/without LPS stimulation detected by ferrozine assays. (C) Ferritin iron staining for EVs from CTRL/LPS‐treated Caco‐2 cells. PC: positive control, commercial iron containing horse ferritin. (D) Uptake efficiency of Cy5‐labelled iron‐loaded ferritin (Holo‐Ft) and iron‐free ferritin (Apo‐Ft) by RAW264.7 cells using flow cytometry and the quantification of the mean fluorescent intensity. (E–J) RAW264.7 cells were treated with ferric ammonium citrate (FAC), iron‐loaded ferritin (Holo‐Ft), and iron‐free ferritin (Apo‐Ft). (E) Levels of iron metabolism, inflammation and oxidative stress‐associated proteins detected by western blotting. (F) Levels of cytosolic labile iron pool. (G) Cytosolic iron contents detected by Ferro Orange probe and the quantification of the relative fluorescence intensity. Bar: 50 µm. (H) ROS levels detected by DCFH‐DA using flow cytometry and the quantification of the mean fluorescence intensity. (I) Malondialdehyde (MDA) levels. (J) mRNA levels of *Il‐6*, *Nlrp3* and *Tnfα* detected by RT‐qPCR. (K) mRNA levels of M1/M2 phenotype markers detected by RT‐qPCR. Values are shown as mean ± SEM. t‐test was used for the comparison between the two groups. One‐way ANOVA was utilized for the comparison among 3 groups. *p < 0.05, **p < 0.01, ***p < 0.001.

We then investigated whether the secreted ferritin is iron‐loaded or not. The results showed a significant increase in iron, as revealed by ferrozine assays (Figure 6A,B) and in‐gel iron staining (Figure 6C). Then, we detected the uptake efficiency of iron‐free Apo‐ferritin and iron‐loaded Holo‐ferritin by macrophages. The results showed that RAW264.7 cells took up these two Cy5‐labelled ferritins with similar efficiency (Figure 6D). To reveal that the iron‐loading into ferritin is critical to promote oxidative stress and inflammation, we compared the effects of iron, Apo‐ferritin (Apo‐Ft), and Holo‐ferritin (Holo‐Ft). The results showed a significant increase in proteins Nlrp3, Sod2, ferroportin (Fpn) and FtH and a decrease in transferrin receptor 1 (Tfr1) following Holo‐Ft treatment (Figure 6E). The iron‐loaded status of ferritin was confirmed by cytosolic LIP assays (Figure 6F) and cellular ferrous iron probe Ferro Orange staining (Figure 6G). However, the similar cellular iron status between FAC and Holo‐groups presented significantly different responses, evidenced by the elevated ROS levels (Figure 6H) in RAW264.7 cells, though a comparable response to FAC and Holo‐Ft in terms of malondialdehyde (MDA) content, a lipid peroxidation indicator (Figure 6I). Accordingly, the inflammatory genes *Il‐6*, *Nlrp3*, and *Tnfα* were upregulated in Holo‐Ft‐treated cells, whereas these effects were not observed in cells treated with either FAC or Apo‐Ft (Figure 6J). To further investigate the polarization state of RAW264.7 cells, we detected the expression of both pro‐ and anti‐inflammatory genes using RT‐qPCR. The results showed unchanged or even decreased expression of anti‐inflammatory genes while significantly increased expression of pro‐inflammatory genes after Holo‐Ft treatment (Figure 6K). These experiments were repeated in human THP‐1 cells, yielding similar results (Figure S9B). These results demonstrate that iron‐loaded ferritin promotes oxidative stress and inflammatory responses in macrophages.

### 3.5 MSR1 is Crucial for the Uptake of Ferritin‐Loaded EVs by Macrophages

**FIGURE 7:**
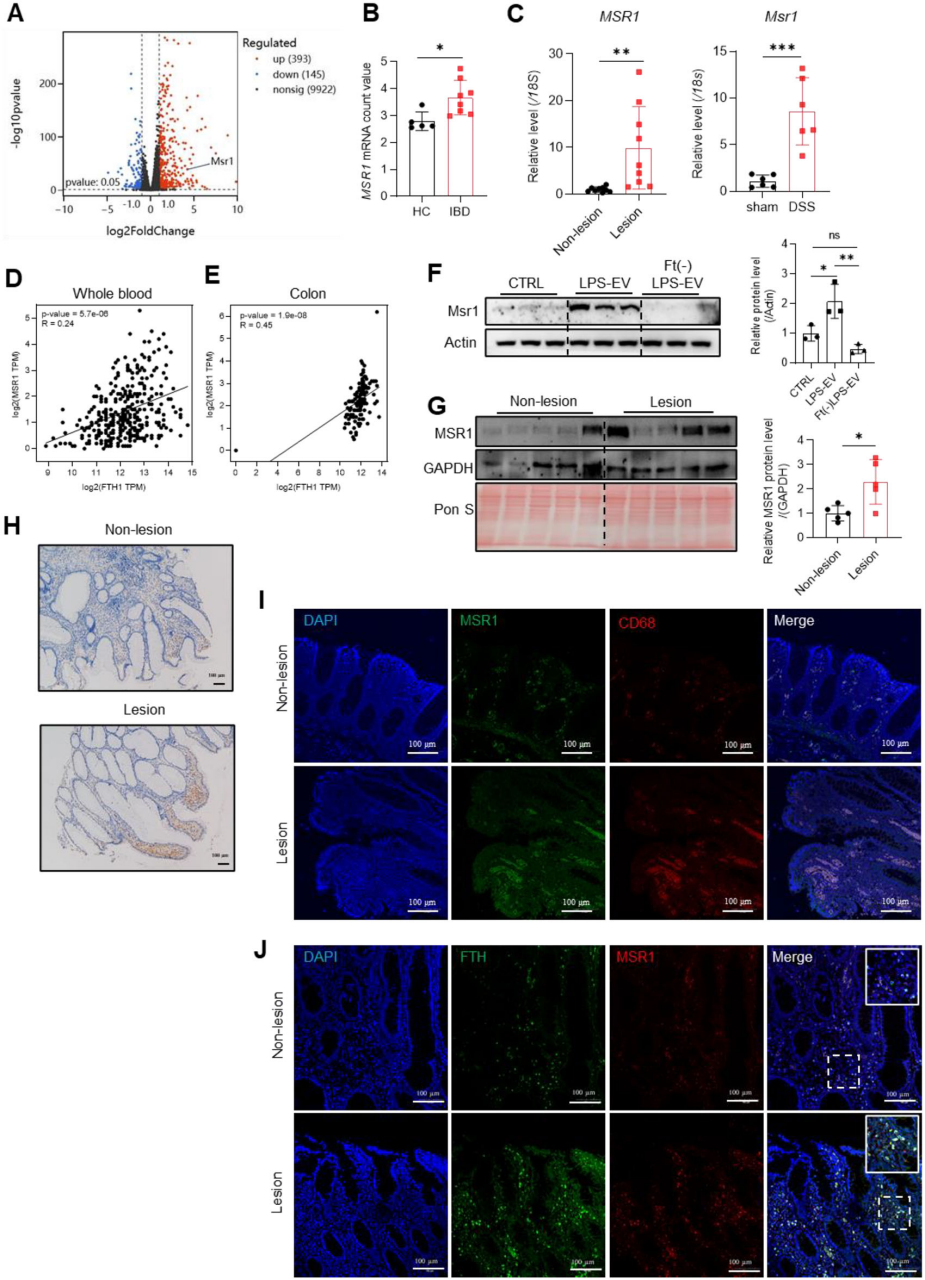
MSR1 is crucial for the ferritin uptake of macrophages. (A) The volcano plot of differentially expressed genes between LPS‐EV and CTRL‐EV group RAW264.7 cells from transcriptomics in this study. (B) *MSR1* mRNA levels in colon mucosa from ulcerative colitis patients and healthy controls. The raw data were from GEO profile GDS3119. (C) *MSR1* mRNA levels in intestinal sections from IBD patients collected in this study and DSS‐induced colitis mice, detected by RT‐qPCR. D‐E: Correlation of *MSR1* and *FTH1* in (D) whole blood and (E) colon. The raw data were from GEPIA database. (F) Representative images and quantification of Msr1 protein levels in RAW264.7 cells treated with EVs from Caco‐2 cells stimulated with LPS (LPS‐EVs) or FtH depleted EVs (Ft(‐) LPS‐EVs). (G–J) Intestinal samples from IBD patients: (G) MSR1 protein levels and the quantification. (H) Representative images of immunohistochemistry for MSR1. Bar: 100 µm. (I) Co‐localization of MSR1 and macrophages. The macrophages were labelled with CD68 and the nuclei were labelled with DAPI. Bar: 100 µm. (J) Confocal images of the FTH (green) and MSR1 (red) in intestinal sections from IBD patients. Nuclei were labelled with DAPI (blue). Bar: 100 µm. Values are shown as mean ± SEM. *t*‐test was used for the comparison between two groups. One‐way ANOVA was utilized for the comparison among 3 groups. *p < 0.05, **p < 0.01, ***p < 0.001.

Next, we asked how macrophages take up the ferritin‐loaded EVs. Previous studies suggest that MSRs, especially MSR1 (also named SCARA1) and SCARA5 can bind ferritin or EVs (Li et al. 2009; Kanno et al. 2020; Yu et al. 2020; Khanam et al. 2021). Intriguingly, we analysed the significantly differentially expressed genes (DEGs) in the transcriptomic data of RAW264.7 cells treated with EVs and found that *Msr1* was one of the top‐ranked genes (Figure 7A), but not other *SCARA* (*SCARA2‐5*). By reanalysing publicly available GEO data (https://www.ncbi.nlm.nih.gov/geo, #GDS3119), we found that *MSR1* expression was upregulated in the colon mucosa of patients with IBD (Figure 7B), while *SCARA2‐5* mRNA levels remained constant (Figure S10A). Further examination of *SCARAs* mRNA levels revealed similar results in intestinal tissues from IBD patients and DSS‐induced colitis mice using RT‐qPCR (Figure 7C and S10B). And a significantly positive correlation between *MSR1* and *FTH1* mRNA levels was identified in whole blood and colon samples by reanalysing the data from the GEPIA database (http://gepia.cancer‐pku.cn/) (Figure 7D,E). This correlation was also observed in RAW264.7 cells, where treatment with ferritin‐loaded EVs upregulated Msr1, unlike treatment with ferritin‐depleted EVs from LPS‐stimulated Caco2 cells (Figure 7F, LPS‐EV *vs*. Ft(‐)LPS‐EV). Immunoblotting and immunohistochemical assays strengthened the observation of increased MSR1 in the intestinal lesion sites of IBD patients (Figure 7G,H), with MSR1 being almost exclusively expressed in macrophages (Figure 7I), These findings support the notion that ferritin‐loaded EVs stimulates inflammation, leading to upregulation of Msr1 in macrophages.

**FIGURE 8:**
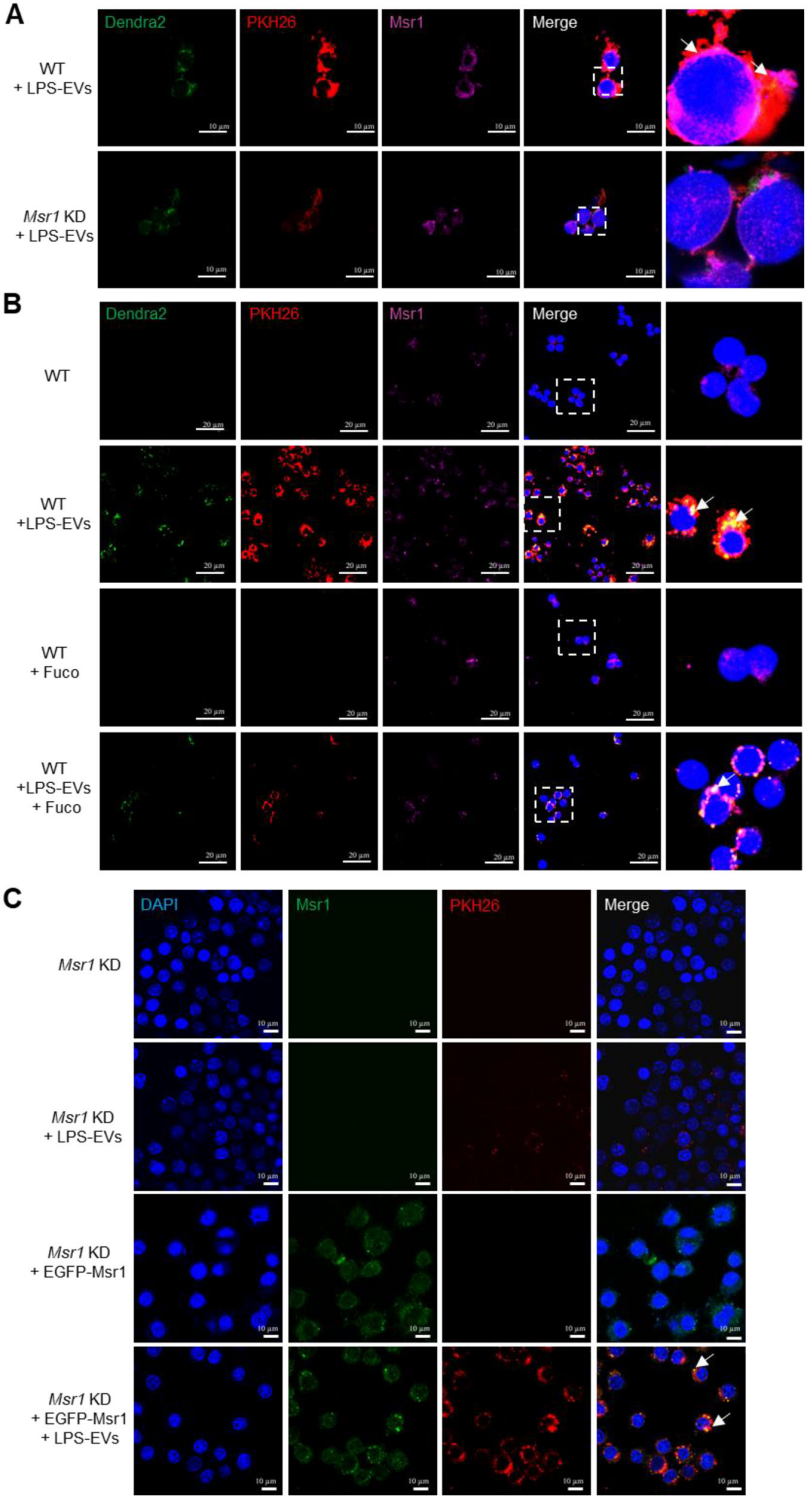
MSR1 is crucial for the EVs internalization to bring ferritin into the macrophages. (A) Confocal images of the Dendra2 (green) and Msr1 (purple) in WT or *Msr1* KD RAW264.7 cells, treated with EVs from Dendra2‐FtH expressing Caco‐2 cells. EVs were labelled with fluorescent probe PKH26 (red). Nuclei were labelled with DAPI (blue). Bar: 10 µm. (B) Confocal images of the Dendra2 (green) and Msr1 (purple) in RAW264.7 cells, treated with EVs from Dendra2‐FtH‐ expressing Caco‐2 cells, with/without fucoidan treatment. EVs were labelled with fluorescent probe PKH26 (red). Nuclei were labelled with DAPI (blue). Bar: 20 µm. (C) Confocal images of EGFP‐Msr1 (green) and EVs (red) in *Msr1* KD RAW264.7 cells, following transfection with pEGFP‐Msr1 plasmid and treatment with PKH26‐labelled EVs. Nuclei were labelled with DAPI (blue). Bar: 10 µm.

To explore whether MSR1 mediates the internalization of IECs‐derived ferritin‐loaded EVs into macrophages, we first confirmed the co‐localization of MSR1 and FTH in inflamed intestinal sections from patients with IBD (Figure 7J). Then, we isolated EVs from Caco‐2 cells expressing Dendra2‐FtH following LPS treatment and labelled the EVs with PKH26. RAW264.7 cells were then cultivated with these double‐fluorescent EVs. We observed that Dendra2‐FtH, PKH26, and Msr1 were well co‐localized in RAW264.7 cells (Figure 8A, wild type [WT]). However, the uptake of EVs and their co‐localization were significantly reduced in *Msr1* KD cells (Figure 8A, lower panel). Additionally, treating RAW264.7 cells with the MSR1 antagonist fucoidan produced similar results (Figure 8B). To further confirm the vital role of MSR1 in uptake of EVs in macrophages, we performed rescue experiments on *Msr1* KD cells by transfecting pEGFP‐Msr1 plasmid. The result showed that pEGFP‐Msr1 expression rescued *Msr1* KD RAW264.7 cells to take up the EVs (Figure 8C). These findings indicate that MSR1 is crucial for the uptake and internalization of ferritin‐containing EVs in response to inflammatory stimulation.

### 3.6 MSR1 Antagonist Fucoidan Mitigates the Severity of DSS‐Induced Colitis

**FIGURE 9:**
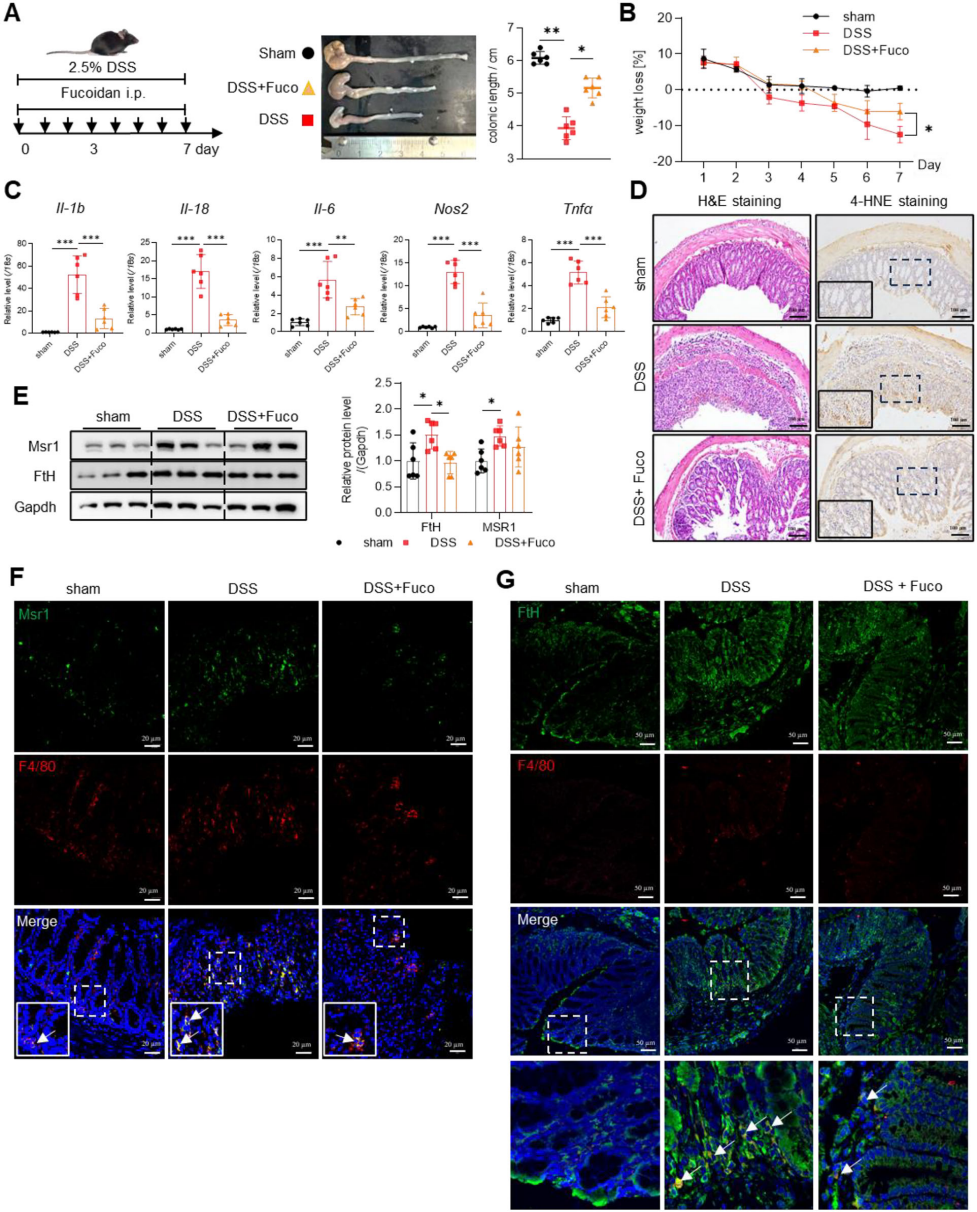
MSR1 antagonist fucoidan mitigates the severity of IBD in DSS‐induced colitis mice. C57BL/6J wildtype mice were given 2.5% DSS drinking water and intraperitoneally injected with fucoidan every day for 7 days. *N* = 6/group. (A) Representative images of colon and quantification of the length of colon. (B) Body weight. (C) mRNA levels of inflammatory genes in colon tissues detected by RT‐qPCR. (D) Representative images of H&E‐stained and 4‐HNE‐stained colon tissues. Bar: 100 µm. (E) FtH and Msr1 protein levels in colon tissue. (F) Representative confocal images in colon tissues. F4/80 is in red, Msr1 in green, and nuclei in blue. Bar: 20 µm. (G) Confocal images showing less FtH in macrophages after fucoidan treatment. Macrophages were labelled in red, FtH in green, nuclei in blue. Bar: 50 µm. Values are shown as mean ± SEM. *t*‐test was used for the comparison between the two groups. One‐way ANOVA was utilized for the comparison among more than 3 groups. *p < 0.05, **p < 0.01, ***p < 0.001, ns: no significance.

To further elucidate the role of MSR1‐mediated ferritin uptake in vivo, we administrated fucoidan to DSS‐induced colitis mice via daily intraperitoneal injections from Day 0 to Day 7. Mice treated with fucoidan exhibited significant protection from colitis, evidenced by longer colonic length, less weight loss, and lower expression levels of inflammatory genes such as *Il‐1b*, *Il‐18*, *Il‐6*, *Nos2*, and *Tnfα* (Figure 9A–C). Histopathological analysis using H&E staining revealed significant improvements in fucoidan‐treated mice (Figure 9D, left), and 4‐HNE staining indicated markedly reduced lipid peroxidation in the colon (Figure 9D, right). DSS treatment significantly increased the protein levels of Msr1 in the lesional colon (Figure 9E), mainly in macrophages (Figure 9F), but fucoidan treatment did not significantly alter Msr1 expression (Figure 9E), in agreement with other studies (Hsu et al. 1998; Tang et al. 2018; Guo et al. 2019). However, F4/80 and its co‐localization with FtH were significantly reduced in the colon following fucoidan administration (Figure 9G), supporting that fucoidan blocks the Msr1‐mediated uptake of ferritin‐loaded EVs in vivo to prevent inflammation.

We also used a colitis‐treatment model, in which mice were given 2.5% DSS in drinking water for 7 days, followed by sterile regular water for another 3 days. Fucoidan was administrated daily from Day 4 to Day 10 (Figure S11A). Mice treated with fucoidan showed better recovery from colitis compared to those given water only, as indicated by longer colonic length, improved survival, reduced weight loss, better histomorphology, and reduced lipid peroxidation (Figure S11A–D). Other tested parameters including Msr1 expression, infiltrated macrophages, and co‐localization of FtH and F4/80 are all similar to that in the prevention model except FtH expression (Figure S11E–G). These results indicate that fucoidan mitigates or improved the outcome of DSS‐induced colitis by disrupting the FtH‐Msr1 axis in mice.

To demonstrate the effects of EVs derived from IECs on the development of IBD, we administered Caco‐2 cell‐derived EVs via intraperitoneal injection in DSS‐induced colitis model, and further intervened with fucoidan treatment (Figure S12A). Same parameters were measured. Overall, LPS‐EV treatment aggravated the symptoms of colitis while fucoidan treatment counteracted the effects from both DSS and LPS‐EVs (Figure S12B–G). These results indicated that EVs from IECs, especially from inflamed IECs, aggravated the severity of IBD. Fucoidan, as MSR1 inhibitor, mitigated the effects of EVs by reducing uptake of EVs and FtH levels in macrophages.

## 4 Discussion

This study demonstrates that the secreted ferritin from IECs via EV pathway contributes to the development of DSS‐induced colitis. The iron‐loaded ferritins within EVs are internalized by macrophages in an MSR1‐dependent manner, leading to iron accumulation, oxidative stress, and inflammatory exacerbation (Figure S13). This observation was corroborated by clinical samples from patients with IBD. Our research reveals that secreted ferritin bridges the interaction between IECs and macrophages, playing a crucial role in the pathogenesis and progression of IBD. Additionally, we have provided evidence for the therapeutic efficacy of fucoidan, which intervenes in the interaction between macrophages and ferritin‐containing EVs. This suggests the potential for combining fucoidan with conventional therapies for enhanced treatment outcomes, particularly for patients with high expression of MSR1.

Iron deficiency anaemia (IDA) is one of the most common metabolic complications of IBD, as a result of chronic blood loss and reduced absorption of iron (Akhuemonkhan et al. 2017). Oral iron supplementation has been known to cause oxidative stress and mucosal damage, which aggravates inflammation and promotes carcinogenesis (Carrier et al. 2002). Both rodent models and human studies have indicated that the generation of ROS due to Fenton reaction potentially leads to further deteriorate IBD (Erichsen et al. 2005; Semrin et al. 2006). Thus, treating IBD patients with IDA with iron supplementation is very cautious in clinic. The treatment to improve IDA is usually prescribed during the remission phase, rather than in the progressive phase of the disease. Our findings support the clinical fact.

The intestinal tract is a complex organ where gut microbiota, epithelial cells, immune cells, and other cell types form an interconnected network functioning as a system (Chen et al. 2022). The merits and demerits of interactions between different cell types rely on what they communicate. We found here that IECs secreted iron‐loaded ferritin via the EV pathway to aggravate macrophage inflammatory response. However, semaphorin 7A expression on the basolateral side of IECs induces macrophages to express IL‐10 to ameliorate DSS‐induced colitis (Kang et al. 2012). When we used high concentration of GW4869 (5 mg/kg, every other day) to treat DSS‐induced mice, the mice died early (not shown). Thus, the impact of the interaction between IECs and macrophages, whether harmful or beneficial, depends on the context and the mediators acting on macrophages.

Ferritin has long been considered an iron storage protein. The presence of ferritin in late lysosomes and EVs from macrophages supports that macrophages can secrete ferritin through secretory autophagy and EVs, two different non‐classical pathways (Truman‐Rosentsvit et al. 2018). The EV membrane protein CD63 is regulated by IRP, similar to ferritin (Yanatori et al. 2021). This positive correlation among iron, ferritin, and CD63 provides a mechanistic basis for the secretion of iron‐loaded ferritin. Inflammation acts as a stimulus for the secretion process. The re‐localization of ferritin from the apical to the basolateral side of the intestine positions the secreted ferritin‐loaded EVs near infiltrating macrophages. Macrophages tend to sequester more iron to exhibit the innate immune functions by engulfing the invaded bacteria (Kuang et al. 2023), apoptotic cells (Sharma et al. 2023), and EVs (Brown et al. 2019). However, a perseveringly inflammatory burden could overwhelm the adaptive responses of macrophages to exert severe inflammatory response. This scenario is observed when IECs secreted ferritin via EVs, contributing to and aggravating the development of DSS‐induced colitis. Therefore, mice with FtH knockout in IECs exhibit resistance to intestinal inflammation, though FtH ablation in IECs disrupts the systemic iron homeostasis and causes modest inflammation even without DSS stimulation (Vanoaica et al. 2010 and this study).

In humoral immunity, macrophages acquire substantial iron to defend against pathogen infection. Our findings indicate that the uptake of iron‐loaded ferritin imposes an additional burden on macrophages, exacerbating intestinal inflammation, consistent with previous study (Xiao et al. 2024). A vicious feedback loop forms between inflammation and the production and secretion of ferritin with more profound iron content in macrophages. Although the previous studies have demonstrated that iron chelation alleviated the DSS‐induced colitis (Wu et al. 2023; Usman et al. 2024), ferritin should be degraded in that case. In addition to iron content, the ratio of FTH to FTL may also be significant. Previous studies have demonstrated that autophagy induces macrophage‐like cells to secrete ferritin through an autophagy‐based unconventional secretory pathway, predominantly composed of FtH (Kimura et al. 2017). We found that secreted ferritins in EVs from IECs were primarily composed of FtH, with FtL at shallow and nearly undetectable levels (Figures 3D and 6A). FtL was not picked up in the proteomic data (Figure S6D). FTL may not promote inflammation due to high serum levels under physiological conditions. This implies that the composition of serum ferritin varies between pathological and physiological conditions. Therefore, the regulatory details of iron content and subunit composition in ferritin secretion need further exploration.

Growing evidence indicates that EVs can be internalized by binding to receptors on cell membranes (Mathieu et al. 2019). After analysis and verification of the multiple receptors, MSR1 was revealed to be predominantly expressed in macrophages in this study. Its expression has been mainly associated with host defence and cancer (Gudgeon et al. 2022). We confirmed that MSR1 plays an essential role in the internalization of EVs in macrophages to facilitate the engulfment of the iron‐loaded ferritin‐containing EVs. It is noted that except MSR1, other potential receptors, including transferrin receptor (Li et al. 2010) and SCARA5 (Li et al. 2009), were downregulated in the context of inflammation (this study). The application of an MSR1 inhibitor fucoidan inhibited the internalization of ferritin‐containing EVs and alleviated inflammatory responses and the severity of IBD. Therefore, macrophage MSR1 acts as the EV receptors to deteriorate the IBD under inflammatory conditions. Previous studies suggested that a few transcription factors including c‐JUN, ets2, AP‐1 and CEBPB may significantly upregulate *MSR1* expression upon binding to the *MSR1* promoter, and these factors are tightly associated with MAPK‐JNK signalling pathways (Wu et al. 1994; Horvai et al. 1995; Mietus‐Snyder et al. 1998). Our RNA‐seq data revealed that the MAPK signalling pathway is top‐ranked enrichment by KEGG analysis (Figure S8D), correlated with upregulation of *c‐JUN*, *ets2*, *Cebpb*, and *Msr1* (Figure S8G). How EV‐coated iron‐loaded FtH upregulates *Msr1* needs further studies.

In summary, we have demonstrated that, under IBD conditions, IECs secreted iron‐loaded ferritins via EVs, which are taken up by macrophages, exacerbating oxidative stress and inflammatory responses. The EVs engulfment is mediated by macrophage MSR1, and blocking either ferritin or MSR1 produces protective effects. These findings highlight the pathophysiological significance of iron metabolism disorders and suggest potential targets for future clinical applications in patients with IBD.

## Author Contributions

**Wenxin Zhang**: investigation (lead), methodology (lead), funding acquisition (supporting), software (equal), validation (lead), visualization (equal), writing – original draft preparation (equal), formal analysis (equal), conceptualization (supporting), data curation (lead), writing – review and editing (equal). **Weichen Dong**: investigation (supporting), methodology (supporting), resources (supporting), software (equal), validation (equal), visualization (equal), formal analysis (supporting), writing – review and editing (supporting). **Chen Cheng**: software (equal), validation (equal), visualization (supporting), formal analysis (supporting), writing – review and editing (supporting). **Hongting Zhao**: data curation (supporting), formal analysis (supporting), methodology (supporting), software (supporting), writing – review and editing (supporting)**. Yutong Liu**: data curation (supporting), formal analysis (supporting), project administration (supporting), writing – review and editing (supporting). **Kristina Zaydel**: formal analysis (supporting), methodology (supporting), writing – review and editing (supporting)**. Liora Frech**: formal analysis (supporting), methodology (supporting), writing – review and editing (supporting)**. Zhiyao Xing**: software (supporting), visualization (supporting), writing – review and editing (supporting). **Wusheng Zhu**: resources (supporting), writing – review and editing (supporting)**. Bing Zhou**: resources (supporting), writing – review and editing (supporting)**. Yi Li**: resources (supporting), writing – review and editing (supporting)**. Esther G. Meyron‐Holtz**: conceptualization (equal), funding acquisition (equal), resources (supporting), supervision (supporting), writing – review and editing (equal). **Kuanyu Li**: conceptualization (equal), funding acquisition (lead), project administration (lead), resources (lead), supervision (lead), writing – original draft preparation (lead), writing – review and editing (equal).

## Conflicts of Interest

The authors declare no conflicts of interests.

## Supporting information




**FIGURE S1** Verification of DSS‐induced colitis mouse model.
**FIGURE S2** Detection of ferritin in various intestinal cell types showing no significant changes after DSS‐induced intestinal inflammation.
**FIGURE S3** Myeloid *FtH* depletion does not significantly aggravate severity of DSS‐induced IBD in mice.
**FIGURE S4** *FtH^Vil/Vil^* mice show decreased intestinal iron and overloaded systemic iron.
**FIGURE S5** In LPS‐treated macrophages, the mRNA and protein levels of FTH increased, but FTH secretion levels showed no significant change.
**FIGURE S6** FTH was enriched in EVs from inflamed intestinal epithelial cells.
**FIGURE S7** The exosomes from epithelial cells are taken up by macrophages, which was greatly enhanced by LPS stimulation.
**FIGURE S8** The transcriptomic data of RAW264.7 cells treated with CTRL‐EVs or LPS‐EVs.
**FIGURE S9** LPS‐EV or holo‐ferritin treatment induces THP‐1 cells to polarize towards a pro‐inflammatory phenotype.
**FIGURE S10** The mRNA levels of macrophage scavenger receptor 2 to 5 remain constant in inflamed intestinal sections from IBD patients and DSS‐treated mice.
**FIGURE S11** MSR1 antagonist fucoidan treatment accelerates the recovery from DSS‐induced colitis in mice.
**FIGURE S12** MSR1 antagonist fucoidan treatment significantly relieves the aggravating effects of LPS‐EVs and DSS‐induction effects on colitis.
**FIGURE S13** Graphic illustration that macrophages engulf intestinal epithelial cell‐derived EV‐coated FtH via MSR1 to promote inflammation in the development of IBD.
**Table S1**. Patient characteristics.
**Table S2**. Information of primers.
**Table S3**. Information of primary antibodies.

## Data Availability

Data availability statement: All materials are available from the authors on reasonable request (likuanyu@nju.edu.cn).
