# Supplementary material for: Macrophages Internalize Epithelial‐Derived Extracellular Vesicles That Contain Ferritin via the Macrophage Scavenger Receptor 1 to Promote Inflammatory Bowel Disease
Source: J Extracell Vesicles. 2025 Jun 23;14(6):e70105. doi: 10.1002/jev2.70105 (PMC12183398; doi:10.1002/jev2.70105)
Supplement: Supplementary file 1 — FIGURE S1 Verification of DSS‐induced colitis mouse model. FIGURE S2 Detection of ferritin in various intestinal cell types showing no significant changes after DSS‐induced intestinal inflammation. FIGURE S3 Myeloid FtH depletion does not significantly aggravate severity of DSS‐induced IBD in mice. FIGURE S4 FtHVil/Vil mice show decreased intestinal iron and overloaded systemic iron. FIGURE S5 In LPS‐treated macrophages, the mRNA and protein levels of FTH increased, but FTH secretion levels showed no significant change. FIGURE S6 FTH was enriched in EVs from inflamed intestinal epithelial cells. FIGURE S7 The exosomes from epithelial cells are taken up by macrophages, which was greatly enhanced by LPS stimulation. FIGURE S8 The transcriptomic data of RAW264.7 cells treated with CTRL‐EVs or LPS‐EVs. FIGURE S9 LPS‐EV or holo‐ferritin treatment induces THP‐1 cells to polarize towards a pro‐inflammatory phenotype. FIGURE S10 The mRNA levels of macrophage scavenger receptor 2 to 5 remain constant in inflamed intestinal sections from IBD patients and DSS‐treated mice. FIGURE S11 MSR1 antagonist fucoidan treatment accelerates the recovery from DSS‐induced colitis in mice. FIGURE S12 MSR1 antagonist fucoidan treatment significantly relieves the aggravating effects of LPS‐EVs and DSS‐induction effects on colitis. FIGURE S13 Graphic illustration that macrophages engulf intestinal epithelial cell‐derived EV‐coated FtH via MSR1 to promote inflammation in the development of IBD. Table S1. Patient characteristics. Table S2. Information of primers. Table S3. Information of primary antibodies. [file JEV2-14-e70105-s001.docx]

Supplementary Materials for

**Macrophages engulf epithelial-derived ferritin via macrophage scavenger receptor 1 to promote inflammatory bowel disease**

Wenxin Zhang *et al.*

Corresponding authors: Kuanyu Li. Email: [likuanyu@nju.edu.cn](mailto:likuanyu@nju.edu.cn);

Esther Meyron-Holtz. Email: Meyron@technion.ac.il

**This PDF file includes:**

Figs. S1 to S13

Tables S1 to S3

References 1

**
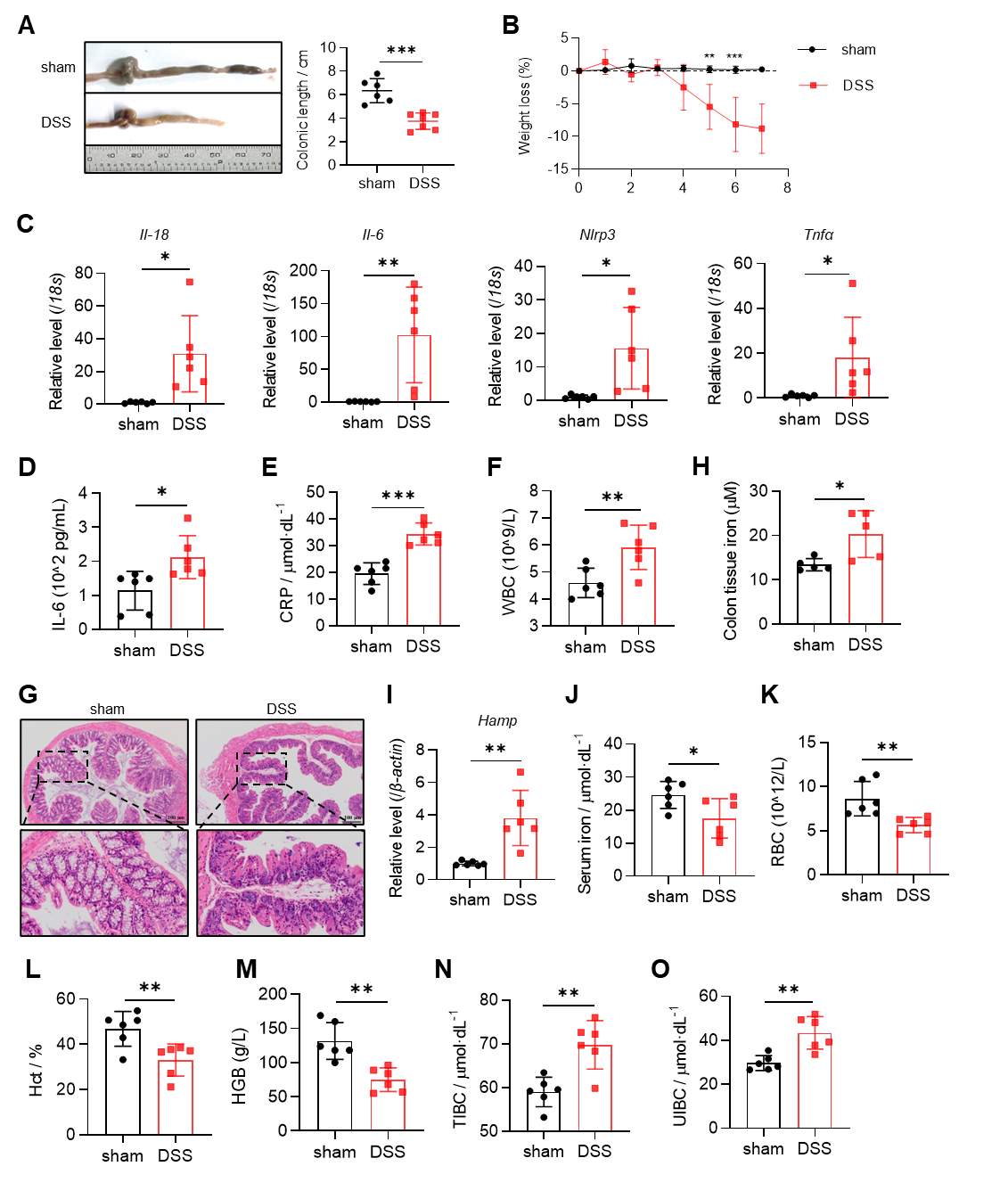
**

**FIGURE S1** Verification of DSS-induced colitis mouse model. C57BL/6J wildtype mice were given 2.5% DSS-drinking water for 7 days. N=6/group. (A) Representative images of colon and quantification of the length of colon. (B) Body weight. (C) mRNA levels of inflammatory genes in colon tissues detected by RT-qPCR. (D) Serum Il-6 levels detected by ELISA. (E) Serum CRP levels detected by ELISA. (F) Count of white blood cells detected by blood routine tests. (G) Representative histologic images of hematoxylin and eosin (H&E)-stained colon tissue slices. Bar: 100 μm. (H) Iron contents in colon by Ferrozine assays. (I) Hepatic *Hamp* levels detected by RT-qPCR; J-O: Indexes from blood tests. (J) Serum iron. (K) Red blood cell count. (L) Hematocrit. (M) Hemoglobin. (N) Total iron binding capacity (TIBC). (O) Unsaturated iron binding capacity (UIBC). Values are shown as mean ± SEM. t-test was used for the comparison between the two groups. *P< 0.05, **P< 0.01, ***P< 0.001, ns: no significance.


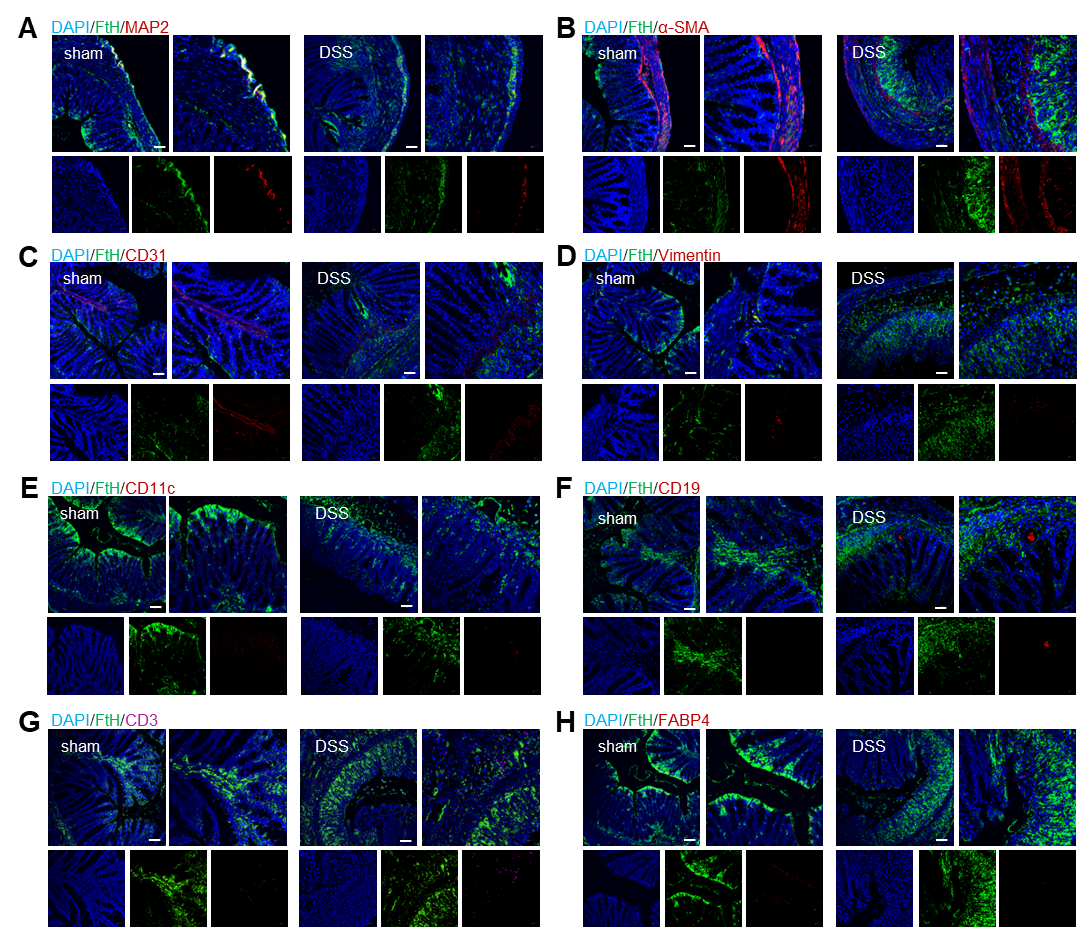


**FIGURE S2** Detection of ferritin in various intestinal cell types showing no significant changes after DSS-induced intestinal inflammation. Representative images of immunofluorescence for FtH (green) and intestinal cell type markers (red) in colon tissue sections. Nuclei: DAPI (blue). Bar: 50 μm. (A) Neurons (MAP2). (B) Smooth muscle cells (α-SMA). (C) Vascular endothelial cells (CD31). (D) Fibroblasts (Vimentin). (E) Dendritic cells (CD11c). (F) B cells (CD19). (G) T cells (CD3). (H) Adipocytes (FABP4).


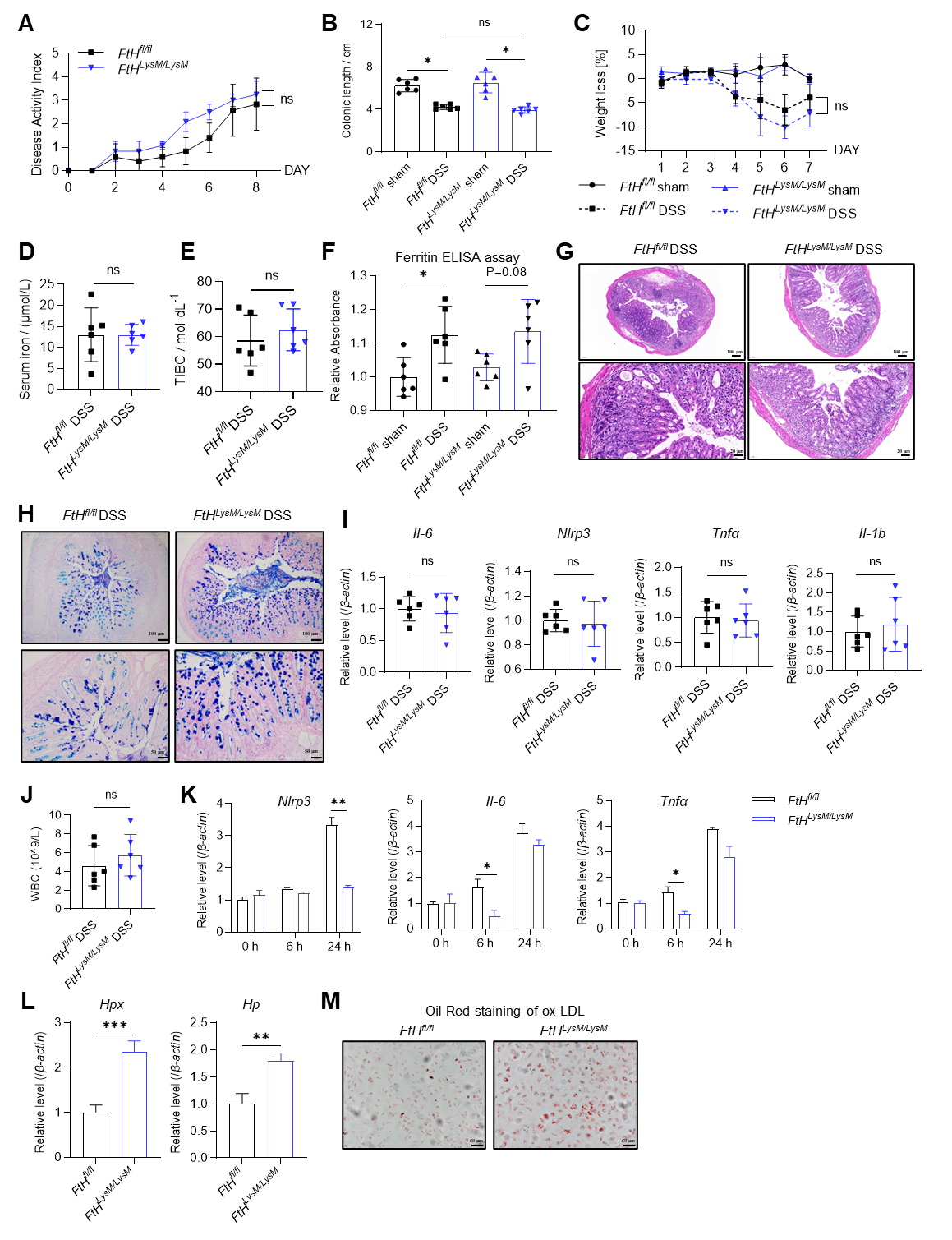


**FIGURE S3** Myeloid *FtH* depletion does not significantly aggravate severity of DSS-induced IBD in mice. *FtH^fl/fl^* and *FtH^LysM/LysM^* mice were given 2.5% DSS drinking water for 7 days. (A) Disease activity index (DAI). (B) Colonic length. (C) Body weight. (D) Serum iron. (E) Total iron binding capacity (TIBC). (F) Serum ferritin detected by ELISA assay. (G) H&E-stained colon tissues. Bar: 100 μm. (H) The alcian blue/periodic acid Schiffs (AB-PAS)-stained colon tissues. Bar: 100 μm. (I) mRNA levels of pro-inflammatory genes of colon tissues detected by RT-qPCR. (J) White blood cell count. K-M: BMDMs were derived from *FtH^fl/fl^* and *FtH^LysM/LysM^* mice. (K) mRNA levels of inflammatory genes in different time points after lipopolysaccharide (LPS) induction detected by RT-qPCR. (L) mRNA levels of receptors for uptake of heme and hemoglobin in macrophages detected by RT-qPCR. (M) Oil red staining of BMDMs treated with ox-LDL for 4 h. Bar: 50 µm. Values are shown as mean ± SEM. t-test was used for the comparison between the two groups. One-way ANOVA was utilized for the comparison among more than 3 groups. *P< 0.05, **P< 0.01, ***P< 0.001, N=6/group, ns: no significance. Not labelled significance means no significance in F and K.


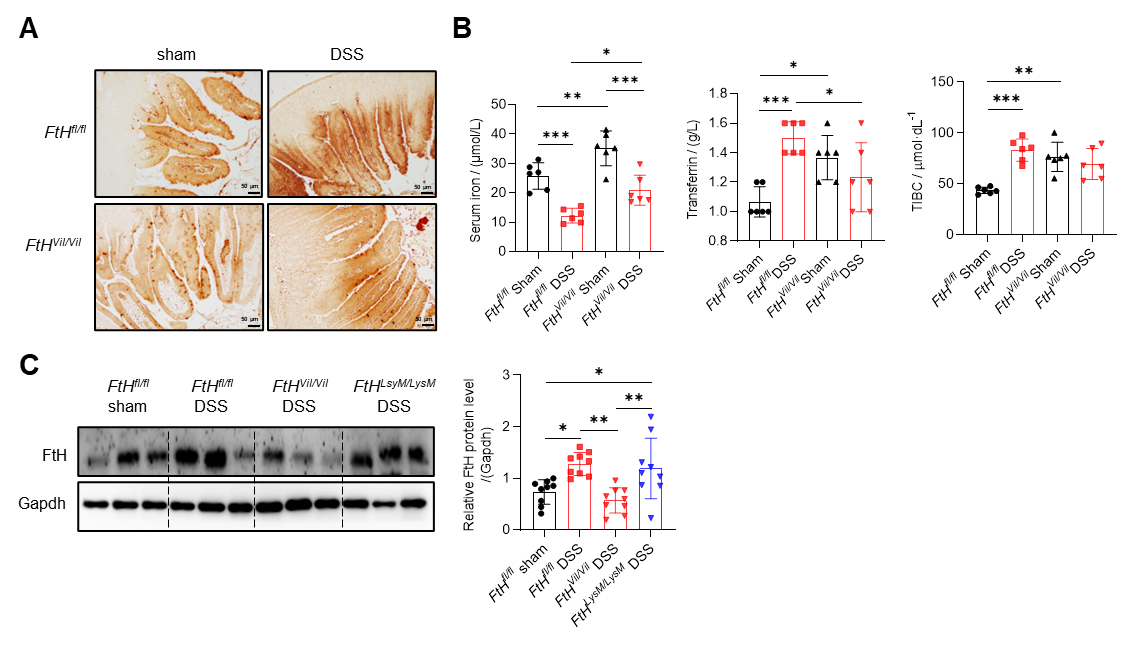


**FIGURE S4** *FtH^Vil/Vil^* mice show decreased intestinal iron and overloaded systemic iron. (A) Diaminobenzidine (DAB)-enhanced Prussian blue iron-stained intestinal sections from *FtH^fl/fl^* and *FtH^Vil/Vil^* mice. Bar: 50 μm. (B) Indexes from serum tests including serum iron, transferrin capacity, and total iron binding capacity (TIBC). (C) FtH protein levels in inflammatory colon of different genotype mice. Quantification sees the right. Values are shown as mean ± SEM. t-test was used for the comparison between the two groups. One-way ANOVA was utilized for the comparison among more than 3 groups. N=6. *P< 0.05, **P< 0.01, ***P< 0.001.


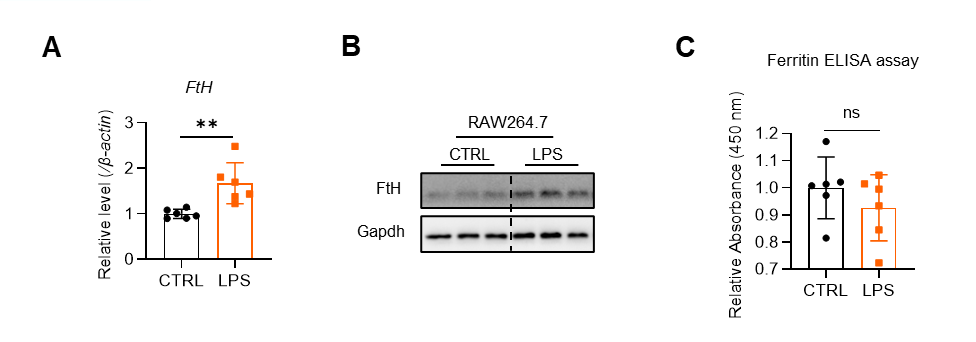


**FIGURE S5** In LPS-treated macrophages, the mRNA and protein levels of FTH increased, but FTH secretion levels showed no significant change. In LPS-treated RAW264.7 cells. (A) mRNA levels of *FtH* detected by RT-qPCR. (B) Protein levels of FtH detected by western blotting. (C) Secreted ferritin detected by ELISA assays. Values are shown as mean ± SEM. t-test was used for the comparison between the two groups. *P< 0.05, ns: no significance.


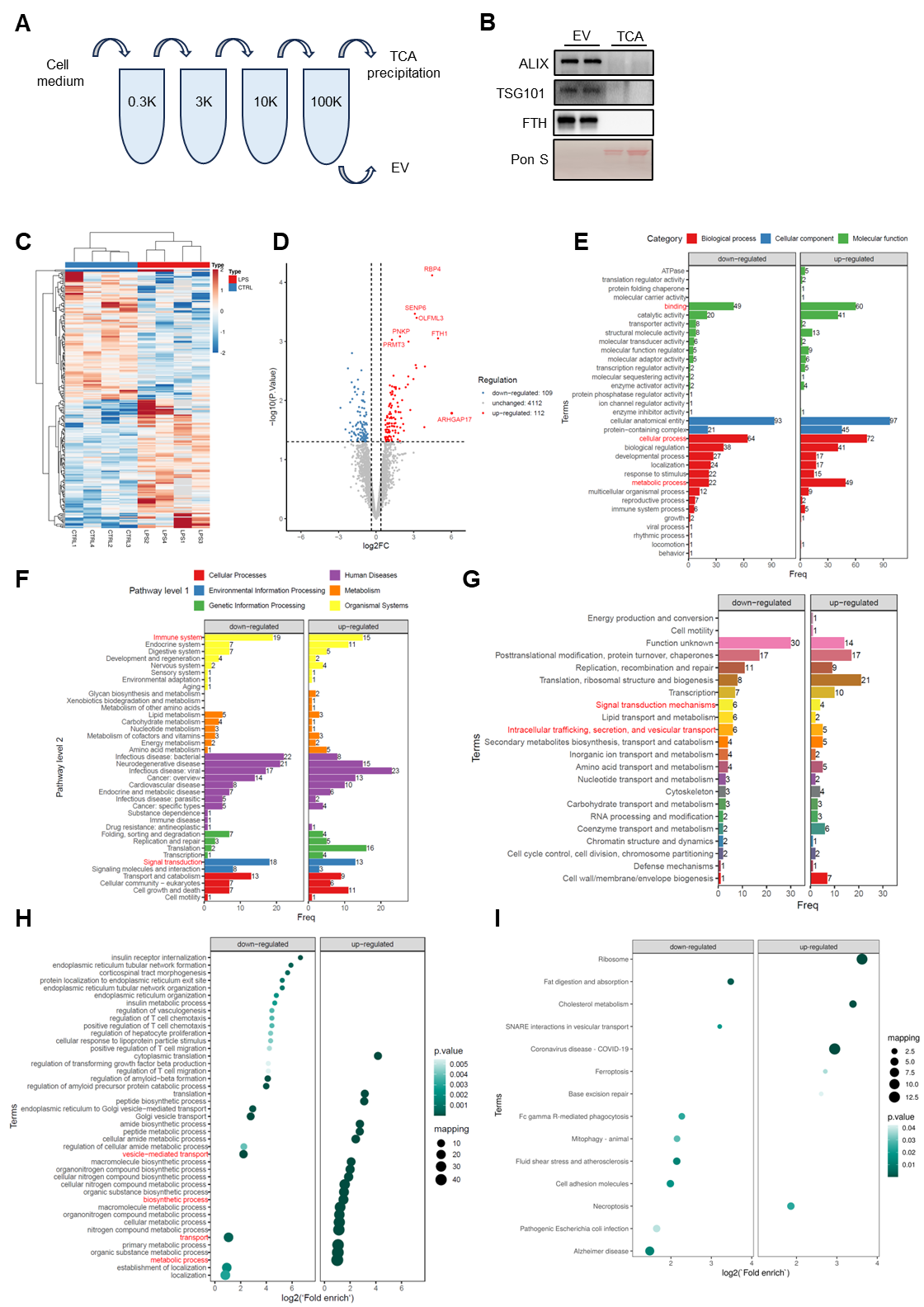


**FIGURE S6** FTH was enriched in EVs from inflamed intestinal epithelial cells. (A) Schematic diagram of the Trichloroacetic acid (TCA) precipitation. (B) The protein levels of FTH in EVs and TCA precipitants. C-I: The proteomic data of EVs from Caco-2 cells with/without LPS stimulation (LPS-EV/CTRL-EV). (C) The heatmap of differential proteins. (D) The volcano plots of differential proteins. (E) GO secondary classification of differential proteins. (F) KEGG pathway classification of differential proteins. (G) COG functional classification. (H) GO enrichment analysis. (I) KEGG pathway enrichment analysis.


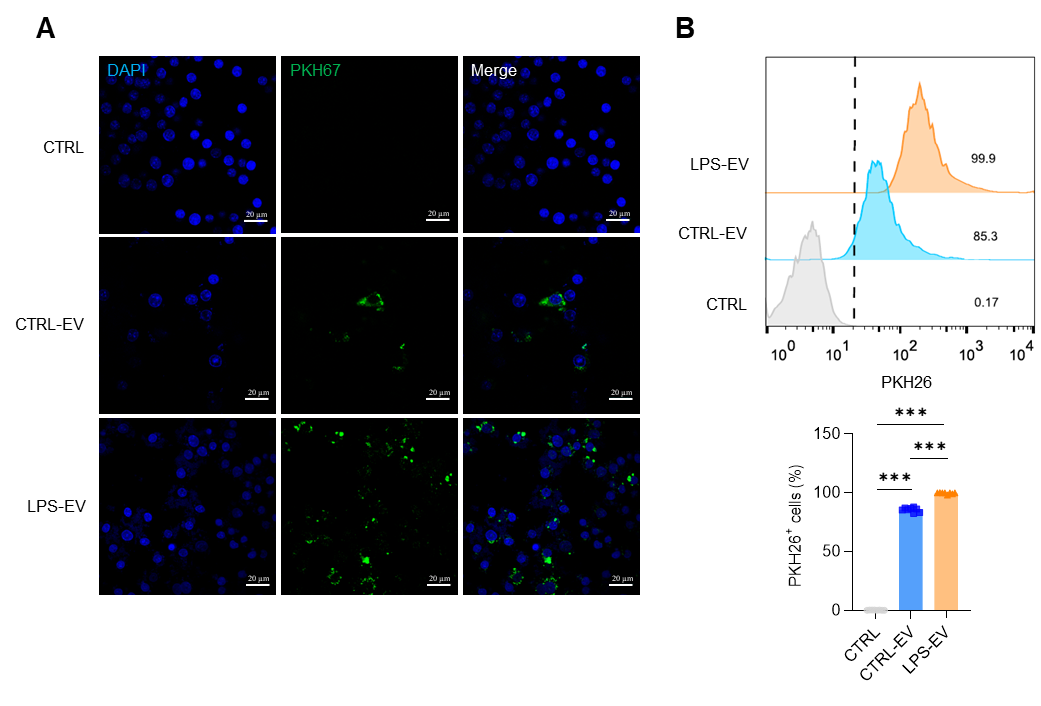


**FIGURE S7** The exosomes from epithelial cells are taken up by macrophages, which was greatly enhanced by LPS stimulation. RAW264.7 cells were cultured with EVs from Caco-2 cells. EVs were labeled with fluorescent probes (PKH67, green in D for fluorescence imaging or PKH26, red in E for Flow cytometry) and nuclei were labeled with DAPI (blue). (A) Representative images of PKH67 fluorescence to show the EVs uptake by RAW264.7 cells. Bar: 20 μm. (B) PKH26 fluorescence intensity detected by flow cytometry and quantification of PKH26^+^ cells. Values are shown as mean ± SEM. t-test was used for the comparison between the two groups. One-way ANOVA was utilized for the comparison among more than 3 groups. *P< 0.05, **P< 0.01, ***P< 0.001.


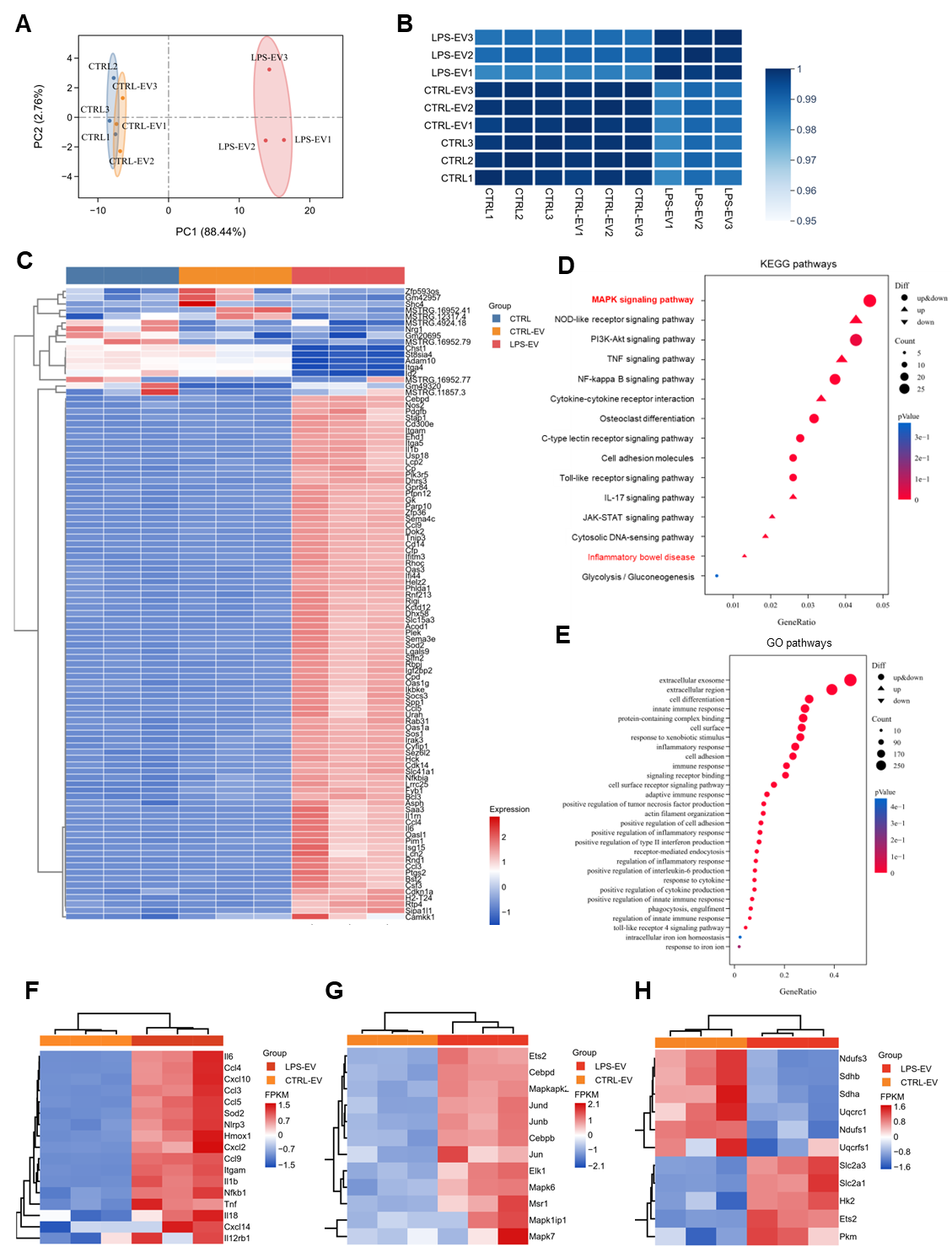


**FIGURE S8** The transcriptomic data of RAW264.7 cells treated with CTRL-EVs or LPS-EVs. (A) The principal component analysis (PCA). (B) The correlation analysis. (C) The multi-group clustering heatmap of differentially expressed genes (DEGs). D-H: LPS-EV *versus* CTRL-EV analysis. (D) KEGG pathway enrichment analysis of DEGs. (E) GO pathway enrichment analysis of DEGs. (F) The expression levels of inflammatory cytokines and chemokines. (G) The expression levels of genes related to MAPK signaling pathway. (H) The expression levels of genes related to glycolysis and oxidative phosphorylation.


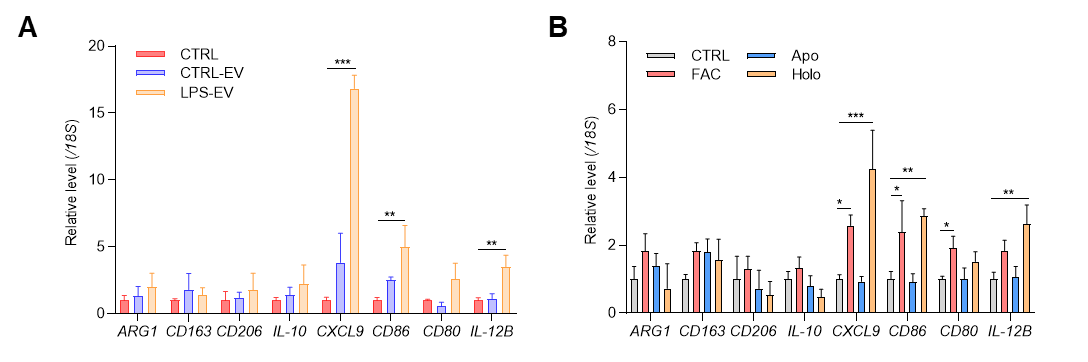


**FIGURE S9** LPS-EV or Holo-ferritin treatment induces THP-1 cells to polarize towards a pro-inflammatory phenotype. THP-1 cells were stimulated with phorbol myristate acetate (PMA) into differentiate to macrophages and then treated with different reagents for 48 h. (A) The expression levels of pro-/anti-inflammatory markers in cells treated with CTRL-EVs or LPS-EVs. (B) The expression levels of pro-/anti-inflammatory markers in cells treated with FAC and Apo- or Holo-ferritin. Values are shown as mean ± SEM. t-test was used for the comparison between the two groups; one-way ANOVA was utilized for the comparison among 3 groups. *P< 0.05, **P< 0.01, ***P< 0.001.


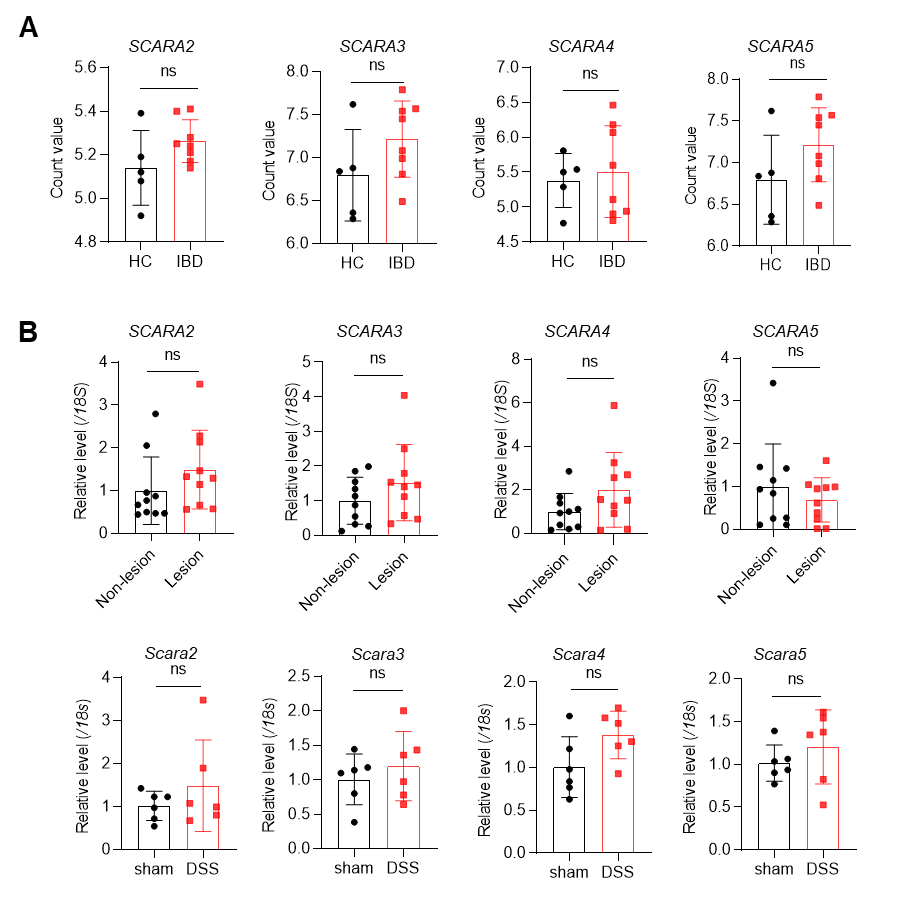


**FIGURE S10** The mRNA levels of macrophage scavenger receptor 2 to 5 remain constant in inflamed intestinal sections from IBD patients and DSS-treated mice. (A) mRNA levels of macrophage scavenger receptor 2-5 in colon mucosa from ulcerative colitis patients and healthy controls. The raw data were from GEO profile GDS3119. (B) mRNA levels of macrophage scavenger receptor 2-5 in intestinal sections from IBD patients or DSS-treated mice detected by RT-qPCR. Values are shown as mean ± SEM. t-test was used for the comparison between the two groups. N=6-10. *P< 0.05, **P< 0.01, ***P< 0.001.

**
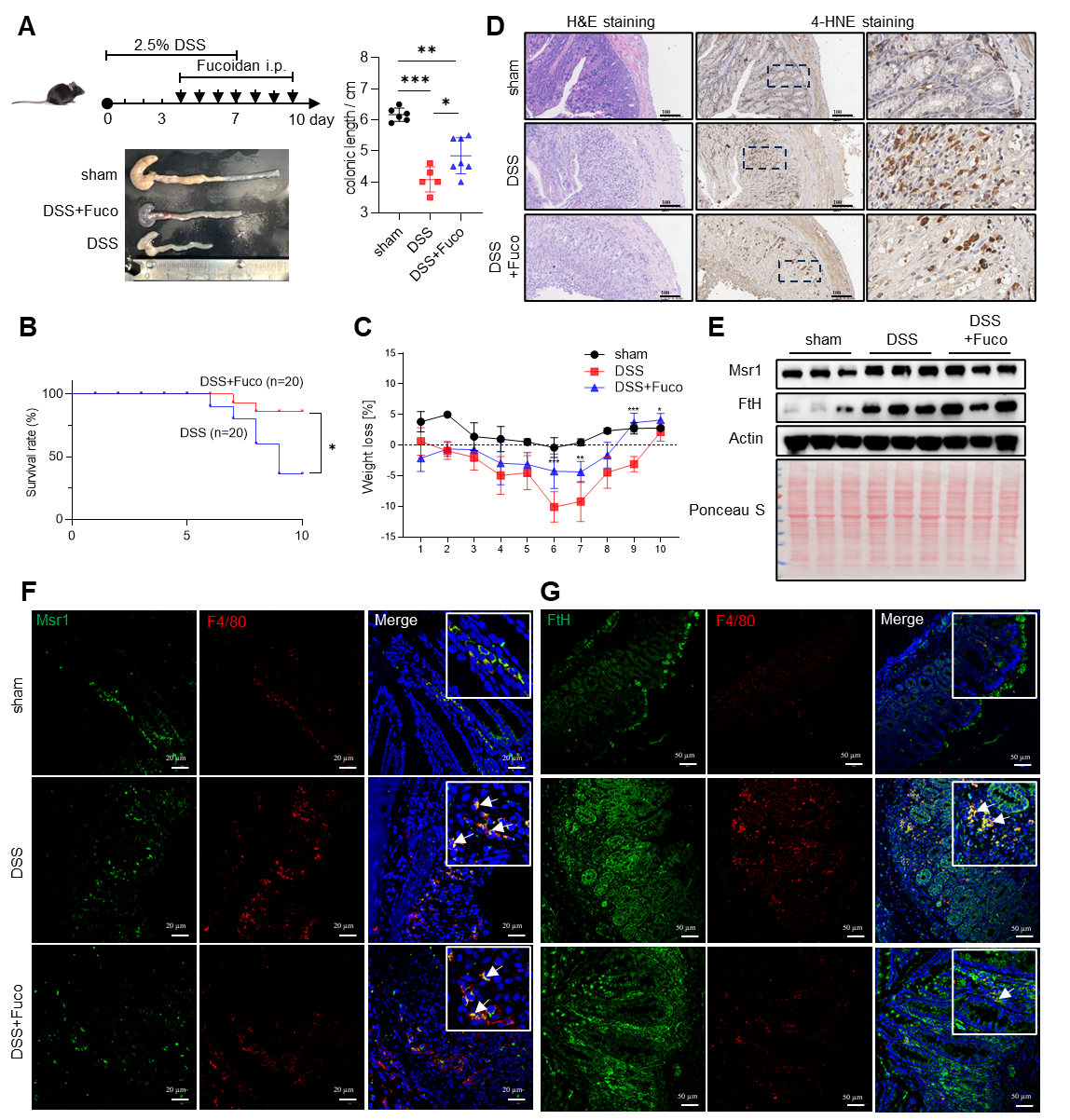
**

**FIGURE S11** MSR1 antagonist fucoidan treatment accelerates the recovery from DSS-induced colitis in mice. C57BL/6J wildtype mice were given 2.5% DSS in drinking water for 7 days and changed into sterile regular water for another 3 days. Fucoidan was intraperitoneal injected daily starting from day 4, when colitis symptoms appeared, to day 10. N=6-20/group. (A) Experimental design scheme and the colon length. (B) Survival rate. (C) Weight loss. (D) Representative images of H&E-stained and 4-HNE-stained colon tissues. Bar: 100 μm. (E) FtH and Msr1 protein levels in colon tissue. (F) Representative confocal images in colon tissues. F4/80 is in red, Msr1 in green, and nuclei in blue. Bar: 20 μm. (G) Confocal images showing less colocalization between FtH and F4/80 after fucoidan treatment. Macrophages were labeled in red, FtH in green, nuclei in blue. Bar: 50 μm. Values are shown as mean ± SEM. t-test was used for the comparison between the two groups. One-way ANOVA was utilized for the comparison among more than 3 groups. *P< 0.05, **P< 0.01, ***P< 0.001, ns: no significance.

**
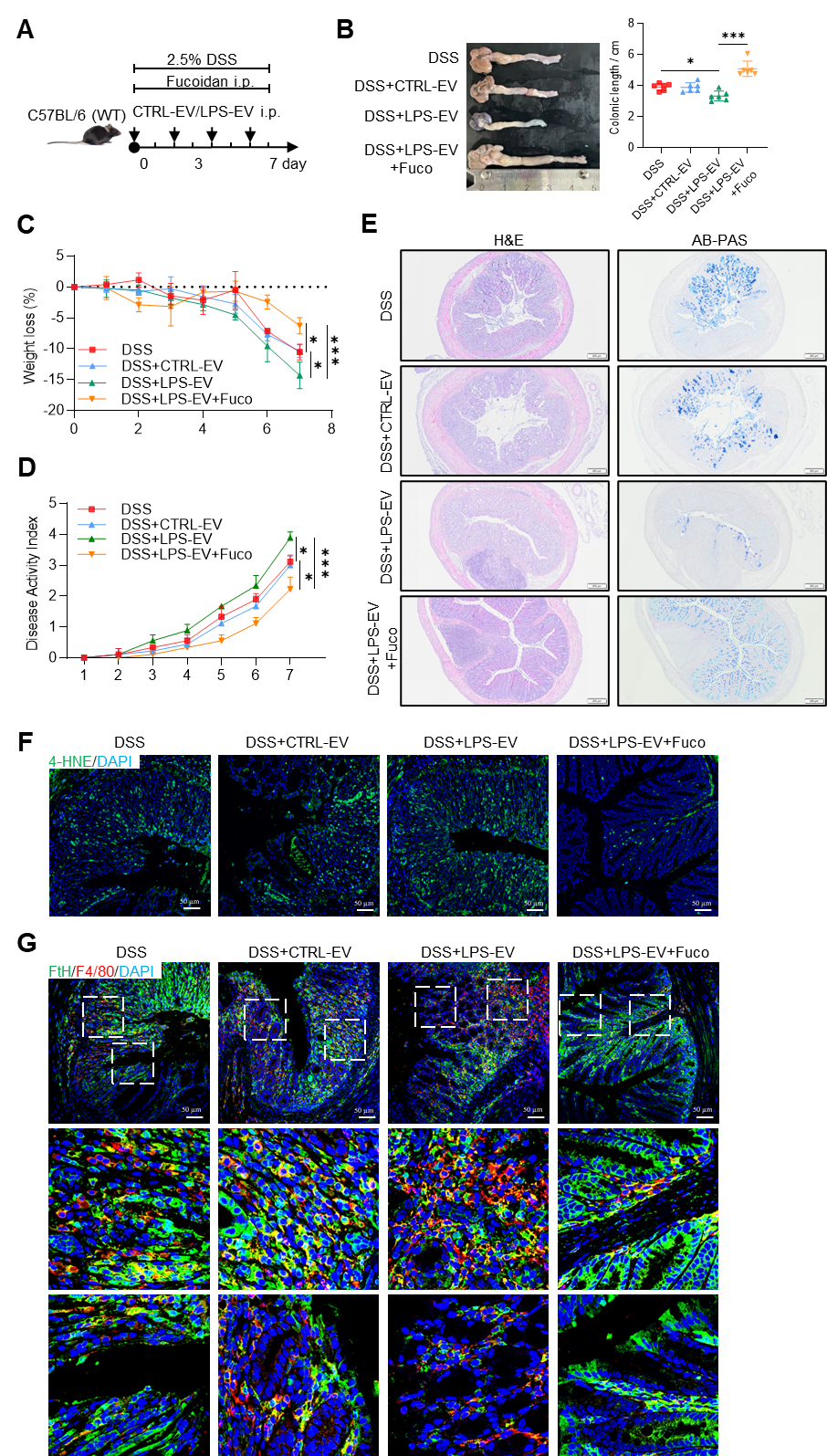
**

**FIGURE S12** MSR1 antagonist fucoidan treatment significantly relieves the aggravating effects of LPS-EVs and DSS-induction effects on colitis. (A) Experimental design scheme. (B) Representative colon length and its quantification. (C) Weight loss. (D) Disease activity index. (E) H&E and AB-PAS staining of colon tissues. Bar: 50 µm. (F) Immunofluorescence showing 4-HNE levels in colon tissues. Nuclei were labeled with DAPI (blue). Bar: 50 µm. (G) Confocal images of FtH (green) and macrophage marker F4/80 (red) in colon tissues. Nuclei were labeled with DAPI (blue). Bar: 50 µm. Values are shown as mean ± SEM. t-test was used for the comparison between the two groups. One-way ANOVA was utilized for the comparison among more than 3 groups. *P< 0.05, **P< 0.01, ***P< 0.001.


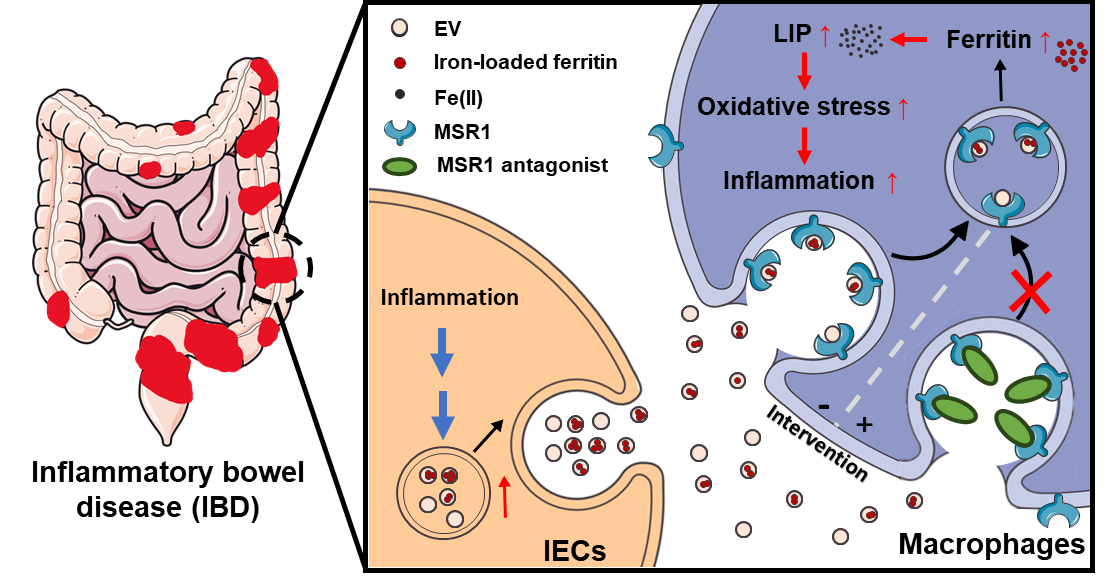


**FIGURE S13** Graphic illustration that Macrophages engulf intestinal epithelial cell-derived EV-coated FtH via MSR1 to promote inflammation in the development of IBD.

**Table S1.** **Patient Characteristics**

| ID | Gender  (M/F) | BMI | Age | Duration disease  (years) | Disease location | Disease behavior | CRP | ALB | ALT | AST | Treatments |
| --- | --- | --- | --- | --- | --- | --- | --- | --- | --- | --- | --- |
| CD1 | M | 26.6 | 60 | 20 | Small intestine | 3 | 4.1 | 44.8 | 12 | 28 | Azathioprine |
| CD2 | M | 17.3 | 27 | 10 | Small Intestine | 3 | 5.5 | 43.2 | 27 | 26 | Adalimumab (anti-TNF) |
| CD3 | M | 18.7 | 18 | 3 | Colon | 3 | 13.6 | 39.1 | 18 | 22 | Azathioprine |
| CD4 | M | 18.2 | 44 | 6 | Colon | 3 | 1 | 43.4 | 31 | 26 | Infliximab (anti-TNF) |
| CD5 | M | 18.0 | 31 | 2 | Colon | 3 | 6 | 45.1 | 63 | 46 | corticosteroids |
| CD6 | M | 20.0 | 65 | 1 | Colon | 3 | 8.4 | 46.5 | 22 | 20 | corticosteroids |
| CD7 | F | 24.2 | 52 | 1 | Small Intestine | 3 | 1.8 | 48.8 | 12 | 20 | Ustekinumab (anti-IL-12) |
| CD8 | F | 16.4 | 26 | 1 | Colon | 3 | 4.7 | 36.5 | 17 | 21 | Infliximab (anti-TNF) |
| CD9 | M | 20.2 | 51 | 3 | Small Intestine | 3 | 3.3 | 35.6 | 31 | 37 | Ustekinumab (anti-IL-12) |
| CD10 | F | 14.4 | 22 | 2 | Colon | 3 | <0.5 | 50.4 | 7 | 16 | Ustekinumab (anti-IL-12) |

CRP: C-reactive protein, mg/L; ALB: albumin, g/L; ALT: alanine aminotransferase, U/L; AST: aspartate transaminase, U/L; Disease behavior: endoscopic score (0: normal; 1: erythema, decreased vascular pattern, mild friability; 2: marked erythema, absent vascular pattern, friability, erosion; 3: spontaneous bleeding, ulcerations).

**Table S2.** **Information of primers**

| gene names | Forward (5'-3') | Reverse (5'-3') |
| --- | --- | --- |
| *18S* | CAGCCACCCGAGATTGAGCA | TAGTAGCGACGGGCGGTGTG |
| *18s* | GGCTACCACATCCAAGGAA | GCTGGAATTACCGCGGCT |
| *ACTIN* | CACCATTGGCAATGAGCGGTTC | AGGTCTTTGCGGATGTCCACGT |
| *Actin* | GCCACTGCCGCATCCTCTTC | AGCCTCAGGGCATCGGAACC |
| *ARG1* | TCATCTGGGTGGATGCTCACAC | GAGAATCCTGGCACATCGGGAA |
| *Arg1* | CATTGGCTTGCGAGACGTAGAC | GCTGAAGGTCTCTTCCATCACC |
| *CD163* | CCAGAAGGAACTTGTAGCCACAG | CAGGCACCAAGCGTTTTGAGCT |
| *Cd163* | GGCTAGACGAAGTCATCTGCAC | CTTCGTTGGTCAGCCTCAGAGA |
| *CD206* | AGCCAACACCAGCTCCTCAAGA | CAAAACGCTCGCGCATTGTCCA |
| *Cd206* | GTTCACCTGGAGTGATGGTTCTC | AGGACATGCCAGGGTCACCTTT |
| *CD80* | CTCTTGGTGCTGGCTGGTCTTT | GCCAGTAGATGCGAGTTTGTGC |
| *Cd80* | CCTCAAGTTTCCATGTCCAAGGC | GAGGAGAGTTGTAACGGCAAGG |
| *CD86* | CCATCAGCTTGTCTGTTTCATTCC | GCTGTAATCCAAGGAATGTGGTC |
| *Cd86* | ACGTATTGGAAGGAGATTACAGCT | TCTGTCAGCGTTACTATCCCGC |
| *CXCL9* | CTAGGCAGGTTTGATCTCCGTTC | TGAACTCCATTCTTCAGTGTAGCA |
| *Cxcl9* | CCTAGTGATAAGGAATGCACGATG | CTAGGCAGGTTTGATCTCCGTTC |
| *FTH1* | AAAGCCATCAAAGAATTGGG | GGGTGTGCTTGTCAAAGAGA |
| *Fth1* | CAAGTGCGCCAGAACTACCA | GCCACATCATCTCGGTCAAAA |
| *Hamp* | CTCCTGCTTCTCCTCCTTGC | GCAATGTCTGCCCTGCTTTC |
| *Icam1* | AAACCAGACCCTGGAACTGCAC | GCCTGGCATTTCAGAGTCTGCT |
| *IL-10* | TCTCCGAGATGCCTTCAGCAGA | TCAGACAAGGCTTGGCAACCCA |
| *Il-10* | CGGGAAGACAATAACTGCACCC | CGGTTAGCAGTATGTTGTCCAGC |
| *IL-12B* | GACATTCTGCGTTCAGGTCCAG | CATTTTTGCGGCAGATGACCGTG |
| *Il-12b* | TTGAACTGGCGTTGGAAGCACG | CCACCTGTGAGTTCTTCAAAGGC |
| *Il-18* | GACTCTTGCGTCAACTTCAAGG | CAGGCTGTCTTTTGTCAACGA |
| *Il-1β* | CAGGCAGGCAGTATCACTCA | AGGCCACAGGTATTTTGTCG |
| *Il-6* | TGAACAACGATGATGCACTTG | CTGAAGGACTCTGGCTTTGTC |
| *MSR1* | TAGGCACTTGGGATGTCTGA | GTCCTCAATTTGTATTGGTGCT |
| *Msr1* | TGGAGGAGAGAATCGAAAGCA | CTGGACTGACGAAATCAAGGAA |
| *Nlrp3* | ATGCTGCTTCGACATCTCCT | AACCAATGCGAGATCCTGAC |
| *Nos2* | ACCTACCGCACCCGAGATG | AAGCCACTGACACTTCGCACA |
| *SCARA2* | GGACAATTTGCGATGACGAGTGG | CCGACACTGAACATTATCCAGCC |
| *Scara2* | ATGGCACCAAGGGAGACAAAGG | GCCTGGTTTTCCAGCATCACCT |
| *SCARA3* | CTCCGAAGACATCTCCTTGACC | CCAGCTTCATGGCAGAAAGAGC |
| *Scara3* | CCACGGAGAAATCCTTCGCAATG | TAGGTCCTCTGCTACCAACAGG |
| *SCARA4* | AGACTCCAAGCATGGTCAGCTC | CCTTTCTGTCCCTTGTTGCCAG |
| *Scara4* | ACTCCAAGCACGGTCAGCTCAT | CTTGTTGCCAGTTGGACCAGGT |
| *SCARA5* | GCTGAACCTGTGTGAGGATGGT | TCAGGAAGACCAGCAGGTAGAG |
| *Scara5* | TGGGAAGCTAGGGGCTACG | CGGCAACATTCAGCTCTCTCT |
| *TFRC* | AGG AAC CGA GTC TCC AGT GA | CTT GAT GGT GCT GGT GAA GT |
| *Tnfα* | ACGTCGTAGCAAACCACCAA | GCAGCCTTGTCCCTTGAAGA |

**Table S3.** **Information of primary antibodies**

| Names | ratio | Company | Cat No. | Use |
| --- | --- | --- | --- | --- |
| FtH | 1:1000 | self-made (Liu, Li et al. 2023) | | WB/IHC/IF |
| GAPDH | 1:5000 | proteintech | 10494-1-AP | WB |
| ACTIN | 1:5000 | proteintech | 23660-1-AP | WB |
| MSR1 | 1:1000 | proteintech | 17858-1-AP | WB |
| TfR1 | 1:1000 | invitrogen | 13-6800 | WB |
| ALIX | 1:1000 | abmart | T57215 | WB |
| TSG101 | 1:1000 | abmart | T55985 | WB |
| E-cadherin | 1:1000 | proteintech | 20874-1-AP | WB |
| Nrf2 | 1:1000 | proteintech | 16396-1-AP | WB |
| Nlrp3 | 1:1000 | abcam | ab263899 | WB |
| Catalase | 1:1000 | proteintech | 21260-1-AP | WB |
| H3 | 1:5000 | abcam | ab1791 | WB |
| HO-1 | 1:1000 | proteintech | 10701-1-AP | WB |
| Sod1 | 1:1000 | proteintech | 10269-1-AP | WB |
| Fpn | 1:1000 | alpha diagnostic | MTP11-A | WB |
| Sod2 | 1:1000 | proteintech | 24127-1-AP | WB |
| CD68 | 1:1000 | abcam | ab125212 | IF |
| F4/80 | 1:200 | abcam | ab6640 | IF |
| MSR1 | 1:1000 | abclonal | A1923 | IF |
| MAP2 | 1:500 | proteintech | 17490-1-AP | IF |
| CD31 | 1:500 | BD Pharmingen | 550274 | IF |
| α-SMA | 1:1000 | proteintech | 14395-1-AP | IF |
| Vimentin | 1:1000 | R＆D systems | MAB2105 | IF |
| CD3 | 1:500 | proteintech | 65060-1-Ig | IF |
| CD19 | 1:500 | proteintech | 27949-1-AP | IF |
| CD11c | 1:500 | proteintech | 60258-1-PBS | IF |
| FABP4 | 1:500 | proteintech | 12802-1-AP | IF |

**References:**

Liu, Y., Y. Li, L. Yang, J. Shen, H. Zhao, W. Dong, Y. Chang, T. Qiao and K. Li (2023). "Stimulation of Hepatic Ferritinophagy Mitigates Irp2 Depletion-Induced Anemia." Antioxidants (Basel) **12**(3).
